# Hippocampal Nogo66‐NgR1 signaling activation restricts postsynaptic assembly in aged mice with postoperative neurocognitive disorders

**DOI:** 10.1111/acel.14366

**Published:** 2024-10-16

**Authors:** Min Jia, Gui‐zhou Li, Jiang Chen, Xiao‐hui Tang, Yan‐yu Zang, Guo‐lin Yang, Yun Stone Shi, Daqing Ma, Mu‐huo Ji, Jian‐jun Yang

**Affiliations:** ^1^ Department of Anaesthesiology, Pain and Perioperative Medicine The First Affiliated Hospital of Zhengzhou University Zhengzhou China; ^2^ Minister of Education Key Laboratory of Model Animal for Disease Study, Model Animal Research Center Nanjing University Nanjing China; ^3^ Department of Anaesthesiology and Perioperative Medicine The First Affiliated Hospital of Nanjing Medical University Nanjing China; ^4^ Perioperative and Systems Medicine Laboratory National Clinical Research Center for Child Health, Children's Hospital, Zhejiang University School of Medicine Hangzhou China; ^5^ Division of Anaesthetics, Pain Medicine & Intensive Care, Department of Surgery & Cancer, Faculty of Medicine Imperial College London, Chelsea & Westminster Hospital London UK; ^6^ Department of Anaesthesiology The Second Affiliated Hospital, Nanjing Medical University Nanjing China

**Keywords:** AMPA receptors, F‐actin depolymerization, hippocampus, Nogo66‐NgR1 signaling, pNCD

## Abstract

Postoperative neurocognitive disorders (pNCD) are a common neurological complication, especially in elderly following anesthesia and surgery. Yet, the underlying mechanisms of pNCD remain elusive. This study aimed to investigate the molecular mechanisms that compromise synaptic metaplasticity in pNCD development with a focus on the involvement of Nogo‐66 receptor 1 (NgR1) in the pathogenesis of pNCD in aged mice. Aged mice subjected to anesthesia and laparotomy surgery exhibited anxiety‐like behavior and contextual fear memory impairment. Moreover, the procedure significantly increased NogoA and NgR1 expressions, particularly in the hippocampal CA1 and CA3 regions. This increase led to the depolymerization of F‐actin, attributed to the activation of the RhoA‐GTPase, resulting in a reduction of dendritic spines and changes in their morphology. Additionally, these changes hindered the efficient postsynaptic delivery of the subunit GluA1 and GluA2 of AMPA receptors (AMPARs), consequently diminishing excitatory neurotransmission in the hippocampus. Importantly, administering the competitive NgR1 antagonist peptide NEP1‐40 (Nogo‐A extracellular peptide residues 1–40 amino acids of Nogo‐66) and Fasudil (a Rho‐kinase inhibitor) effectively mitigated synaptic impairments and reversed neurocognitive deficits in aged mice following anesthesia and surgery. Our work indicates that high hippocampal Nogo66‐NgR1 signaling disrupts postsynaptic AMPA receptor surface delivery due to F‐actin depolymerization in the pathophysiology of pNCD.

## Graphical abstract

Hippocampal Nogo66‐NgR1 signaling activation, disrupting postsynaptic AMPA receptor surface delivery due to F‐actin depolymerization, leads to anxiety‐like behavior and contextual fear memory impairment in aged mice following anaesthesia and surgery.

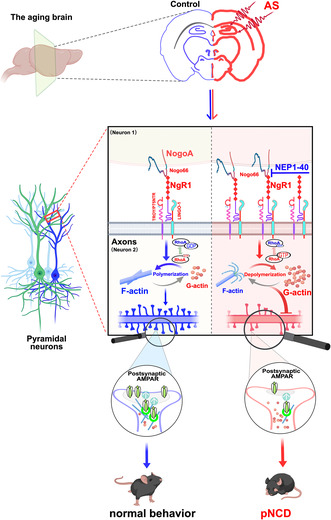

**Abbreviations**
AMPARs: AMPA receptors
AS: anaesthesia and surgery
EZM: elevated zero‐maze test
F‐actin: filamentous actin
FC: fear conditioning
fEPSPs: field excitatory postsynaptic potentials
G‐actin: globular‐actin
GPI: glycophosphatidylinositol‐anchored protein
HFS: high‐frequency stimulation
Jasp: Jasplakinolide
LINGO1: leucine‐rich repeat and immunoglobulin domain‐containing 1
LTP: long‐term potentiation
MAG: myelin‐associated glycoprotein
MAIs: myelin‐associated inhibitors
MBT: marble burying test
NEP1‐40: Nogo‐A extracellular peptide residues 1‐40 amino acids of Nogo‐66
NgR1: Nogo‐66 receptor 1
Nogo: neurite outgrowth inhibitor
NogoA: neurite outgrowth inhibition protein A
OFT: open field test
OMgp: oligodendrocyte glycoprotein
PFC: prefrontal cortex
P75NTR: neurotrophin receptor P75
pNCD: postoperative neurocognitive disorders
POCD: postoperative cognitive dysfunction
POD: postoperative delirium
PPF: paired‐pulse facilitation
PSD: postsynaptic density
RhoA‐GTP: small‐GTPases
TEM: transmission electron microscope
TROY: orphan receptor of tumor necrosis factor‐α receptor superfamily member 19
YMT: Y maze test

## 1 INTRODUCTION

Postoperative neurocognitive disorders manifest significant alterations in emotion, consciousness, and cognitive capability following anesthesia and surgery with a high prevalence in the elderly (Evered et al., 2018; Vacas et al., 2021). Postoperative cognitive dysfunction (POCD) and postoperative delirium (POD) (Alam et al., 2018; Jin et al., 2020) are common phenotypes of postoperative neurocognitive disorders (pNCD). With the increasing aging population, more surgeries are given to the elderly, and surgical complications including pNCD are likely to increase. However, the pathogenesis of pNCD remains largely undefined. While abnormalities of synaptic function and neuronal activity are implicated in contributing to the development of pNCD, little is known about the abnormality of the key regulators that govern balancing synapse stability and plasticity mediating this complication occurrence.

Emerging evidence indicates that Nogo‐66 receptor 1 (NgR1) is pivotal in selectively regulating dendritic spine dynamics, restricting synapse numbers, and orchestrating synaptic assembly for brain plasticity (Akbik et al., 2013; McGee et al., 2005). NgR1 is primarily expressed on the soma and axons of neurons in the central nervous system (CNS) (Wang et al., 2002), which interacts with three myelin‐associated inhibitors (MAIs)—neurite outgrowth inhibitor (Nogo), oligodendrocyte glycoprotein (OMgp), and myelin‐associated glycoprotein (MAG) (Wang, Miao, et al., 2021, Wang, Qin, et al., 2021). Notably, Nogo has three splice variants: NogoA, B, and C, with the latter two being extensively expressed outside the CNS (McKerracher & Winton, 2002). NgR1 interacts with Aβ and mediates the inhibitory effects of Aβ on new synapse assembly and synaptic metaplasticity (Zhao et al., 2017). Study has shown that NgR knockdown reduced amyloid plaque deposition and Aβ levels as well as improved spatial learning and memory in the APP/PS1 mouse model of Alzheimer's disease (AD) (Wang, Miao, et al., 2021, Wang, Qin, et al., 2021). Furthermore, deletion of NgR1 can enhance anatomical changes of synapse markers in post‐traumatic stress disorder and may serve as a therapeutic target for anxiety disorders (Bhagat et al., 2016). These findings suggest that NgR1 signaling potentially holds a crucial part in the development of neurodegenerative diseases.

The proper functioning of the brain in associative learning tasks depends on the modification and improvement of experience‐driven adaptations in synaptic strength. This process involves a dynamic interplay between Hebbian forms of synaptic plasticity and homeostatic synaptic scaling, which is crucial for effective learning and memory (Toyoizumi et al., 2014). At excitatory synapses, one extensively studied form of metaplasticity is postsynaptic synaptic scaling, primarily involving AMPA receptors (AMPARs) (Diering & Huganir, 2018). AMPARs, composed of heterotetrameric combinations of GluA1‐4 subunits, play a major role in excitatory glutamatergic transmission. For instance, in CA1 pyramidal neurons, commonly used as a model for studying long‐term potentiation (LTP), the majority of receptors present are GluA1/GluA2 heteromers, with a minor contribution from GluA2/GluA3 receptors in baseline conditions (Diering & Huganir, 2018). Among these heteromers, GluA1/GluA2 subunits are responsible for synaptic activity–associated transmission, whereas GluA2/ GluA3‐containing receptors maintain a steady synaptic pool of AMPARs constitutively and are rapidly replaced by GluA1‐containing types in an activity‐dependent manner (Henley & Wilkinson, 2016). The regulated abundance and delivery of AMPARs to the synapse are essential steps in modifying synaptic strength, particularly in LTP (Buonarati et al., 2019). Their activity‐dependent trafficking and synaptic transmission are key to synaptic plasticity and metaplasticity events. Dendritic spines, small protrusions on the dendritic shaft of principal neurons, serve as the structural foundation for synaptic plasticity and brain networks, responding to external stimuli. The structure and function of these spines are governed by the remodeling of the actin cytoskeleton, which is crucial for memory stabilization post‐learning (Lamprecht, 2014). Actin, existing in monomeric globular‐actin (G‐actin) and polymerized filamentous actin (F‐actin) forms, maintains a dynamic equilibrium crucial for various aspects of dendritic spine morphology. In dendritic spines, the enriched F‐actin undergoes dynamic modulation, playing a vital role in spine formation, elimination, and remodeling (Luo, 2002). In the research on neurocognitive deficits, synaptosomal F‐actin depolymerization was found to lead to long‐lasting alterations of dendritic spine morphology and density and induced synaptic remodeling impairments (Kommaddi et al., 2018; Li et al., 2023). Hence, preserving the F‐actin to G‐actin ratio within dendritic spines is vital for optimal synaptic functioning. During associative learning, actin dynamics, especially the rapid turnover of actin filaments, regulate the availability of postsynaptic receptors (Gu et al., 2010; Rust et al., 2010). Given that NgR1 signaling is known to influence actin cytoskeleton dynamics during synapse assembly (Mironova & Giger, 2013; Oh et al., 2023; Zhao et al., 2017), our study aimed to investigate whether and how Nogo66‐NgR1 signaling contributes to synaptic plasticity and neurocognitive impairment in aged mice after anesthesia and surgery.

## 2 MATERIALS AND METHODS

This part was briefly described below and the full details are presented as an Appendix S1.

### 2.1 In vivo experiments

#### 2.1.1 Animals

The experimental protocol was approved by the Ethics Committee of Zhengzhou University, Zhengzhou, China and all experimental procedures strictly followed the guidelines of the Animal Care and Use Committee of the National Institutes of Health (Bethesda, MD, USA). C57BL/6 male mice, aged between 8 weeks and 20–22 months, were obtained from Aniphe Biolaboratory Inc. (Nanjing, China). To facilitate their adaptation to the experimental environment, the mice underwent daily handling for 5 min over three consecutive days before the commencement of experiments. Newborn Sprague–Dawley (SD) rat pups (postnatal day 0) for hippocampal neuronal culture experiments were sourced from Changzhou Cavens Lab Animal Co. Ltd. (Changzhou, China). Throughout the experiments, all mice were housed in a 12:12 light/dark cycle, temperature maintained at 22 ± 1°C, and 50 ± 10% humidity with free access to food and water.

#### 2.1.2 Laparotomy surgery

The aged mice (20–22 M old) were randomly assigned to either the control group (without anesthesia and surgery) or the anesthesia and laparotomy surgery group as previously described (Wei et al., 2023).

#### 2.1.3 Cannula placement

A cannula was implanted into the left ventricle of the brain in aged mice under surgical anesthesia 24 h before anesthesia and surgery and connected with the osmatic minipumps for treatment with NgR1‐neutralizing antagonist peptide NEP1‐40 (TAT‐NEP1‐40 TFA form, HY‐P5754A, MCE, Shanghai, China) or the vehicle (98% PBS and 2% DMSO) at a rate of 0.25 μL/min for 9 consecutive days. Mice were randomly assigned to one of four groups based on their treatment: control + vehicle (Con + Vehicle), control + NEP1‐40 (Con + NEP1‐40), anesthesia + surgery + vehicle (AS + Vehicle), and isoflurane anesthesia + laparotomy surgery + NEP1‐40 (AS + NEP1‐40). Another cohort of aged mice, 2 weeks before anesthesia and surgery were implanted with a stainless‐steel guide cannula and ceramic fiber optic ferrules under anesthesia. The accurate placement of the cannula was verified with Nissl staining.

#### 2.1.4 Drug treatment

In this study, NEP1‐40 was applied to counteract the activation of NgR1 caused by the binding of the Nogo66 domain; Fasudil (HY‐10341A, MCE, Shanghai, China) was administrated to investigate the impact of RhoA‐GTPase activity on the pathophysiology in aged mice after anesthesia and surgery, which was injected intraperitoneally at a dosage of 20 mg/kg per mouse. The injections were given once a day starting from 1 h before AS and continued until the completion of the last behavioral test. Mice were randomly assigned to two groups based on their treatment: control + fasudil (Con + Fasudil) and anesthesia + surgery + NEP1‐40 (AS + Fasudil).

#### 2.1.5 Behavioral tests

The behavioral assessments were conducted from 2 to 7 days after surgery in the following order: marble burying (de Brouwer et al., 2019), elevated zero‐maze (Yasumoto et al., 2021), open field (Kraeuter et al., 2019a), Y‐maze (Kraeuter et al., 2019b), and fear conditioning (Shoji et al., 2014) (details see Appendix S1). Data from these tests were recorded using a computer‐operated video tracking system obtained from Shanghai Softmaze Information Technology Co. Ltd. in Shanghai, China. To prevent any potential odor interference from previously tested mice, the testing chambers were meticulously sanitized between each trial using either an MB‐10 solution or a 30% ethanol solution.

#### 2.1.6 In vivo fiber photometry calcium recording

The fiber optic cannula in the right hippocampal CA3 region was implanted under surgical anesthesia 2 weeks before the virus (rAAV9‐hSyn‐GCaMP6s) injection. The GCaMP6 signals were recorded by a photoreceiver system.

#### 2.1.7 Ex vivo sample assessments

After the above experiments, their brain samples were harvested for transmission electron microscope (TEM) to visualize the ultrastructure of neuronal synapses in the hippocampal CA1 stratum radiatum; for Nissl staining to verify the correct placement of cannulas; for Golgi staining with the FD Rapid GolgiStain™ Kit (FD Neurotechnologies, Inc., Columbia, MD, USA) to label the dendrites and spines of neurons (Havekes et al., 2016; Liu et al., 2023); for filamentous (F)‐ and globular (G)‐ actin in the hippocampus with G‐actin/F‐actin in vivo assay kit (BK037, Cytoskeleton, CO, USA); for co‐immunoprecipitation (Co‐IP) to investigate the expression of binding complexes between NgR1 and NogoA at day 7 after anesthesia and surgery with or without treatment with NEP1‐40; for isolation of hippocampal synaptosome with the Syn‐PER™ synaptic protein extraction reagent manual (87793, ThermoFisher Scientific, Shanghai, China).

#### 2.1.8 RhoA pull‐down activation analysis and qRT‐PCR

The fresh hippocampus was harvested for active RhoA (RhoA‐GTPase) detected with a specific RhoA Activation Assay Kit (ab211164, Abcam, Cambridge, UK) and for qRT‐PCR measuring expression of *NgR1*, *NogoA*, *MAG*, and *OMgP* mRNA in the hippocampus, prefrontal cortex (PFC), amygdala, neocortex, striatum, and cerebellum both the control group and the surgery group at 1, 3, and 7 days after anesthesia and surgery, and *Nov*, *Wfs1*, *Iyd*, *Spock1*, *Dsp*, and *Tiam1* mRNA in the hippocampal CA1, CA3, and DG regions in the control aged mice.

### 2.2 Ex vivo experiments

#### 2.2.1 Acute hippocampal slice for electrophysiology

The field excitatory postsynaptic potentials (fEPSPs) and paired‐pulse facilitation (PPF) (Jia et al., 2023; Li et al., 2022) were measured using acute transverse hippocampal slices from adult male C57BL/6 mice (8 weeks old). To simulate neuroinflammation similar to that observed in aged mice after AS, intracerebroventricular injections of LPS (5 μg/2 μL, L4391, sigma) or saline (2 μL) were administered to the adult mice. For the intervention experiments, we applied Nogo‐P4, NEP1‐40, and jasplakinolide to the hippocampal slices through the perfusion line in ACSF. A concentric bipolar stimulating electrode was placed in the stratum radiatum of the CA1 region to stimulate the Schaffer collaterals. LTP and PPF ratio were calculated as the average response recorded 50–60 min after stimulation.

### 2.3 In vitro experiments

#### 2.3.1 Primary culture of hippocampal neurons

Primary hippocampal neuronal cultures were dissociated from SD neonatal rat neonates at postneonatal day 1 following the procedures outlined in our previous study (Li et al., 2022). *Neuron*/*microglia transwell cocultures*: The neuron/microglia cocultures were conducted as previously reported (Chang et al., 2024).

In addition, samples from in vivo and in vitro experiments were homogenized and quantified for Western blotting as described or kept intact for immunofluorescence staining following the protocols described in our previous study (Li et al., 2022) or for hippocampal membrane protein measurements with a surface biotinylation pull‐down method (Nair et al., 2021).

### 2.4 Statistical analysis

Data were presented as mean ± SEM together with a dot plot and then analyzed with one‐way analysis of variance (ANOVA) followed by Bonferroni or Tukey's post hoc test or two‐way ANOVA followed by Tukey's post hoc test as appropriate. The Mann–Whitney test or Student *t* test was used for comparisons between the two groups as appropriate. All analyses were performed using GraphPad Prism 9.0. A *p*‐value of less than 0.05 was considered to be of statistical significance.

## 3 RESULTS

### 3.1 Hippocampal Nogo66‐NgR1 expressions persistently increased in CA1 and CA3 regions in aged mice after anesthesia and surgery

**FIGURE 1:**
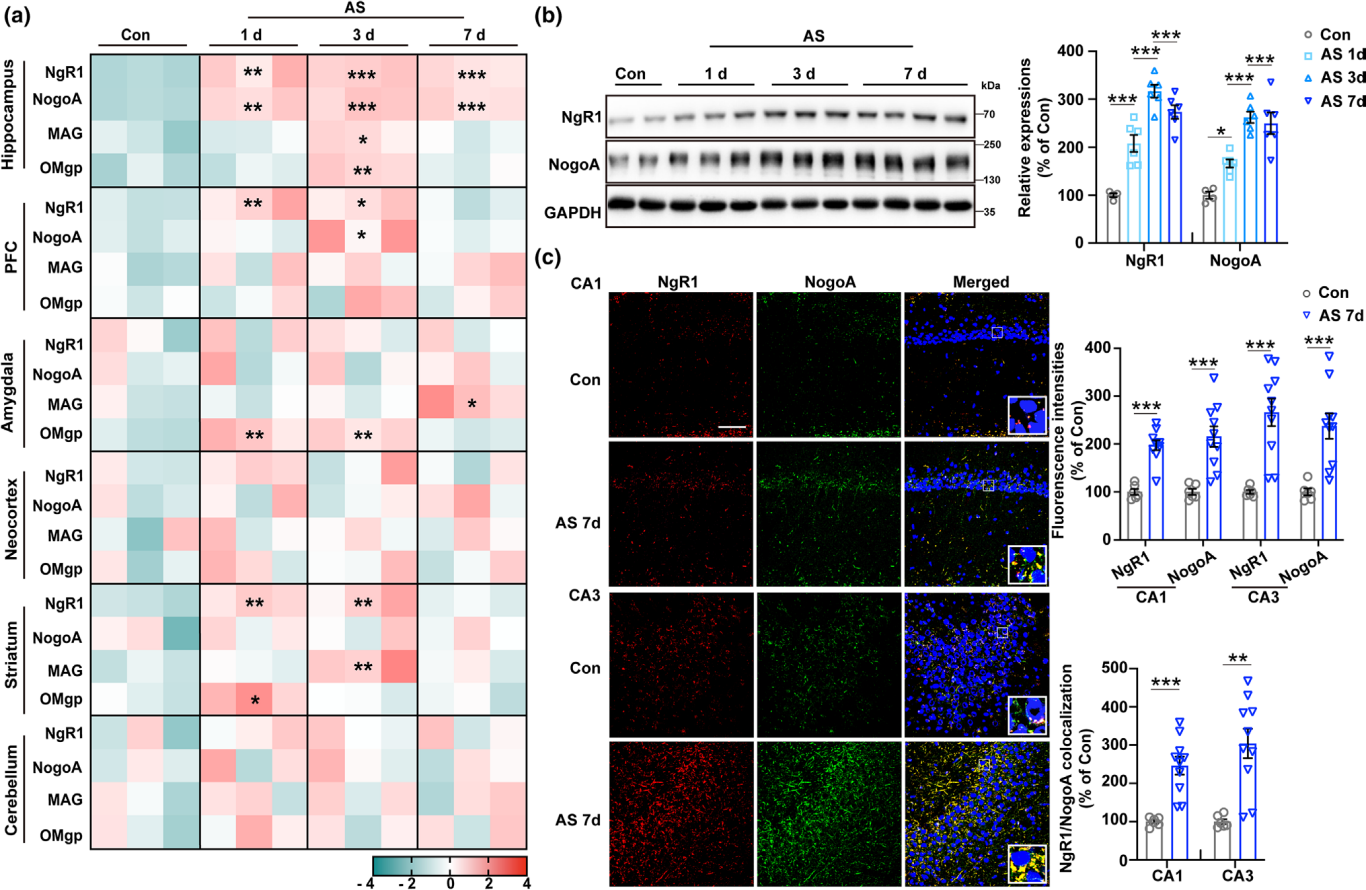
Persistent overexpression of NgR1 and NogoA in hippocampal subregions CA1 and CA3 of aged mice following anesthesia and surgery (AS). (a) The transcript enrichment levels of NgR1, NogoA, MAG, and OMgp were assessed using qRT‐PCR in the hippocampus, PFC, amygdala, neocortex, striatum, and cerebellum at different time points in aged mice with or without AS (*n* = 3/group). (b) The expressions of NgR1 and NogoA in the hippocampus were examined at different time points in aged mice, both with and without AS (*n* = 4 of the Con group, *n* = 6 of the groups of AS 1, 3, and 7 d). (c) Immunofluorescence (IF) images of NgR1 and NogoA in the hippocampal CA1 and CA3 subregions of aged mice from the Con and AS 7 d groups were captured at a magnification of 40×. The relative fluorescence intensities of NgR1, NogoA, and the level of their colocalization were analyzed (*n* = 6 of the Con group, *n* = 10 of the AS 7 d group). Scale bar = 50 μm. (a, b) Comparisons were made with one‐way ANOVA followed by Turkey's post hoc test. (c) The two groups were compared using an unpaired *t* test with the Mann–Whitney U test. All data were presented as mean ± SEM; *p < 0.05, **p < 0.01, and ***p < 0.001 compared with the Con group. AS, anesthesia and laparotomy surgery; Con, control.

To assess the expressions of NgR1‐associated signaling pathways in the brains of aged mice after anesthesia and surgery, we examined the mRNA levels of NgR1, NogoA, MAG, and OMgp in some emotion and cognition‐associated regions, with a particular focus on the hippocampus, PFC, amygdala, neocortex, and striatum together with cerebellum. The expressions were analyzed using qRT‐PCR at different time points following anesthesia and surgery (AS 1, 3, and 7 d) compared to the control group. In the hippocampus, the mRNA levels of NgR1 and NogoA at postanesthesia and surgery days 1, 3, and 7 were consistently increased when compared to those of the control group (Figure 1a). Subsequently, the protein levels of NgR1 and NogoA in the hippocampus were also significantly increased at postanesthesia and surgery days 1, 3, and 7 compared to that of the controls (Figure 1b). Since NgR1 is a glycophosphatidylinositol‐anchored protein (GPI) lacking an intracellular component, it necessitates transmembrane coreceptors to facilitate signal transduction from the extracellular environment to the cytosol. Hence, we also examined the expressions of NgR1 signal‐transducing partners, including LINGO1, TROY, and P75NTR, in the hippocampus. The expressions of LINGO1, TROY, and P75NTR were found to be concurrently increased at 1, 3, and 7 days after anesthesia and surgery compared to the control group (Figure S1a).

The relative fluorescence intensities of NgR1, NogoA, and the level of their colocalization in the hippocampal CA1 and CA3 regions were detected increasingly (Figure 1c), while no significant change was observed in the DG region (Figure S1b) after 7 days of anesthesia and surgery compared to the control group. Moreover, the precise isolation of the hippocampal subregions with specific markers was confirmed for the CA1, CA3, and DG regions (Figure S1c). Subsequently, the NgR1 expression at day 7 after anesthesia and surgery compared to the aged control was significantly increased in the CA1 and CA3 regions relative to the DG region compared to the control group (Figure S1d).

### 3.2 NgR1 competitive antagonistic peptide NEP1‐40 treatment alleviated anxiety‐like behavior and contextual fear memory in aged mice after anesthesia and surgery

**FIGURE 2:**
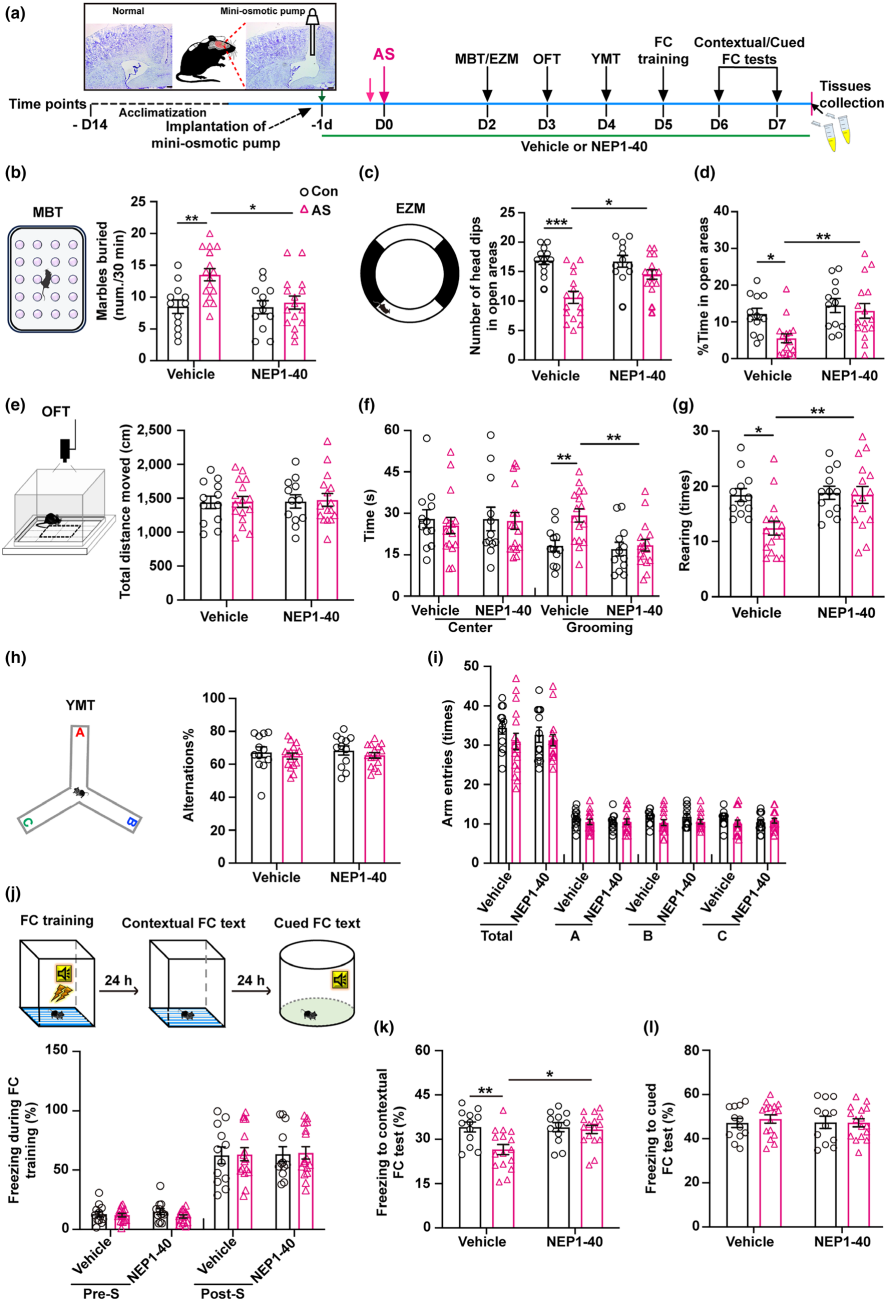
Efficacy of NgR1 antagonistic peptide NEP1‐40 in rescuing anxiety‐like behavior and contextual fear memory impairments in aged mice following anesthesia and surgery. (a) A diagrammatic presentation of the experimental protocol was provided, illustrating the schedule of drug treatment, behavioral tests, and brain harvest following the implantation of mini‐osmotic pumps into the lateral ventricles. Nissl staining was performed to confirm the accurate positioning of the osmotic cannula. Scale bar = 200 μm. (b) The schematic representation of the marble burying test (MBT) was displayed. The number of marbles buried within a 30‐min period was recorded and compared among the four groups. (c, d) The diagram of the elevated zero maze (EZM) was depicted. A comparison of the number of head dips and the percentage of time spent in the open areas during a 5‐min session was conducted among the four groups. (e–g) The diagram of the open field test (OFT) was illustrated. To compare the behavior among the four groups, various parameters were recorded during a 5‐min session in the chamber including total distance moved, time spent in the center and grooming, as well as the number of rearing events. (h, i) The diagrammatic representation of the Y‐maze test (YMT) was provided. The percentage of alternations and the number of arm entries during the 8‐min session were analyzed for each of the four groups. (j–l) The diagrammatic drawing of the fear conditioning (FC) test was presented. The percentage of freezing during the FC training, contextual, and cued FC tests were analyzed separately for each of the four groups. (b–l) Data were analyzed using 2‐way ANOVA (2 × 2 factors: Groups × treatments) followed by Turkey's post hoc test and were presented as mean ± SEM; *n* = 12 of the Con + Vehicle and Con + NEP1‐40 groups, and *n* = 16 of the AS + Vehicle and AS + NEP1‐40 groups. *p < 0.05 and **p < 0.01. AS, anesthesia and surgery; Con, control.

To investigate the impact of hippocampal Nogo66‐NgR1 signaling on neurocognitive disorders in aged mice after anesthesia and surgery, we utilized NgR1 competitive antagonist peptide NEP1‐40 (Nogo‐A extracellular peptide residues 1–40 amino acids of Nogo‐66) to specifically block the binding of Nogo66 to NgR1 via i.c.v. administration with micro‐osmotic pump infusion of the experimental diagram (Figure 2a). Aged mice following anesthesia and surgery exhibited an increased number of buried marbles in the MBT (Figure 2b), a decreased number of head dips (Figure 2c), and a reduced percentage of time spent in the open arms (Figure 2d) in the EZM. Additionally, they displayed increased time spent in grooming (Figure 2f) and decreased times of rearing in the OFT (Figure 2g), as well as a decreased percentage of freezing during the contextual FC test (Figure 2k), when compared to the control + vehicle group. However, the administration of NEP1‐40 reversed all these behavioral changes. No significant differences were observed among the four groups in terms of total distance moved (Figure 2e) and time spent in the center in the OFT (Figure 2f), as well as the percentage of alternations (Figure 2h) and times of arm entries (Figure 2i) in YMT. Additionally, there were no notable distinctions in the percentage of freezing time during the FC training session (Figure 2j) and the cued FC test (Figure 2l) among the four groups.

### 3.3 Alleviation of anesthesia and surgery‐induced postsynaptic damage in aged mice through NEP1‐40 treatment

**FIGURE 3:**
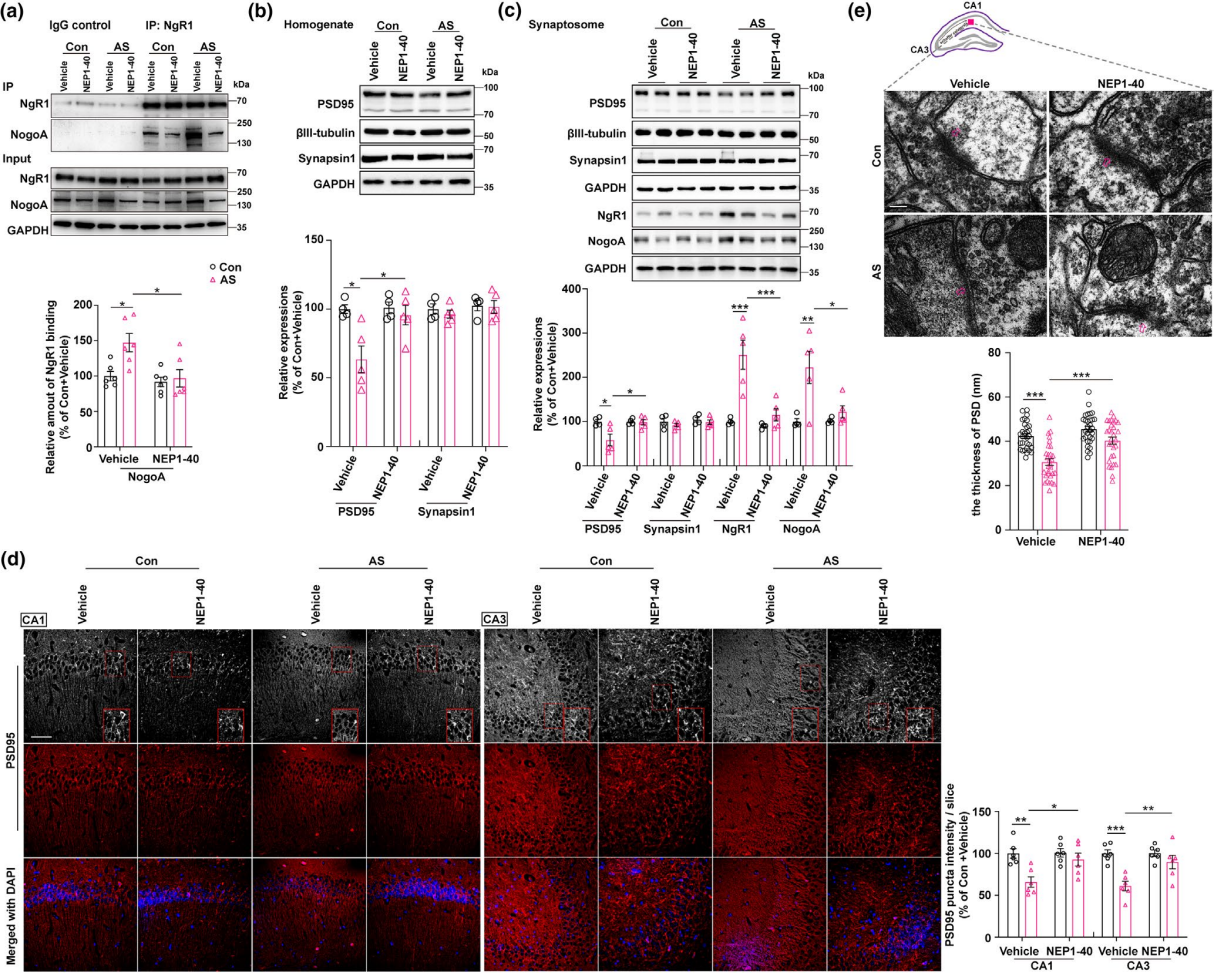
Effects of AS and NEP1‐40 treatment on hippocampal postsynaptic changes in aged mice. (a) The co‐immunoprecipitation (CO‐IP) assay revealed the binding interaction between NgR1 and NogoA following AS or NEP1‐40 treatment, in comparison to the Con + Vehicle group (*n* = 6/group). (b) Expressions of postsynaptic marker PSD95 and presynaptic marker Synapsin1 were assessed by western blot (WB) in hippocampal homogenate from the four groups (*n* = 4 of the Con + Vehicle and Con + NEP1‐40 groups; *n* = 5 of the AS + Vehicle and AS + NEP1‐40 groups). (c) Expressions of PSD95, Synapsin1, NgR1, and NogoA were detected by WB assay in hippocampal synaptosome among the four groups (*n* = 4 of the Con + Vehicle and Con + NEP1‐40 groups; *n* = 5 of the AS + Vehicle and AS + NEP1‐40 groups). (d) Immunofluorescence (IF) staining was conducted to visualize the expression of PSD95 in hippocampal CA1 and CA3 subregions across the four groups (*n* = 6 of each group). (e) Transmission electron microscope (TEM) images were utilized to observe alterations in the thickness of the postsynaptic density (PSD) within the stratum radiatum of the hippocampal CA1 region across the four groups. A total of ten synapses per mouse were selected for measuring the PSD thickness; *n* = 30 synapses (10 synapses/mice). (a–e) Data were analyzed by 2‐way ANOVA (2 × 2 factors: Groups × treatments) followed by Turkey's post hoc test. Data were represented as mean ± SEM. *p < 0.05, **p < 0.01, and ***p < 0.001.

The CO‐IP analysis revealed a noticeable reduction in the formation of binding complexes between NgR1 and NogoA in the hippocampus after NEP1‐40 treatment (Figure 3a). It is known that NgR1 plays a critical role in restricting synaptogenesis and synaptic plasticity, which are essential for enhancing synaptic efficiency and facilitating emotion and associative learning tasks. Thus, our study examined the expression levels of the postsynaptic marker PSD95 and presynaptic marker synapsin1 in both the homogenate and synaptosome of hippocampal CA1 and CA3 regions' lysate. Compared to the control + vehicle group, the anesthesia and surgery + vehicle group exhibited a significant reduction of the expression of PSD95 in both the homogenate (Figure 3b) and synaptosome (Figure 3c). However, this reduction was reversed following NEP1‐40 treatment as opposed to the anesthesia and surgery + vehicle group (Figure 3b,c). Nevertheless, the expression of presynaptic synapsin1 in the hippocampal homogenate and synaptosome remained unchanged among the four groups (Figure 3b,c). Moreover, the levels of NgR1 and NogoA in the hippocampal synaptosome were elevated in the anesthesia and surgery + vehicle group compared to the control + vehicle group. However, the NEP1‐40 treatment normalized these levels (Figure 3c). To comprehensively assess the postsynaptic damage, we employed immunofluorescence staining and transmission electron microscope techniques to identify and examine the postsynaptic alterations. In comparison to the control + vehicle group, we observed a decrease in the intensities of PSD95 puncta within the hippocampal CA1 and CA3 regions (Figure 3d). Additionally, there was a reduction in the thickness of PSD in the stratum radiatum of the hippocampal CA1 region (Figure 3e). However, these effects were effectively reversed with NEP1‐40 treatment (Figure 3d,e).

### 3.4 Restoration of hippocampal F‐/G‐actin equilibrium and alleviation of dendritic spine remodeling through NEP1‐40 treatment by limiting RhoA‐GTPase hyperactivation in anesthesia and surgery‐exposed aged mice

**FIGURE 4:**
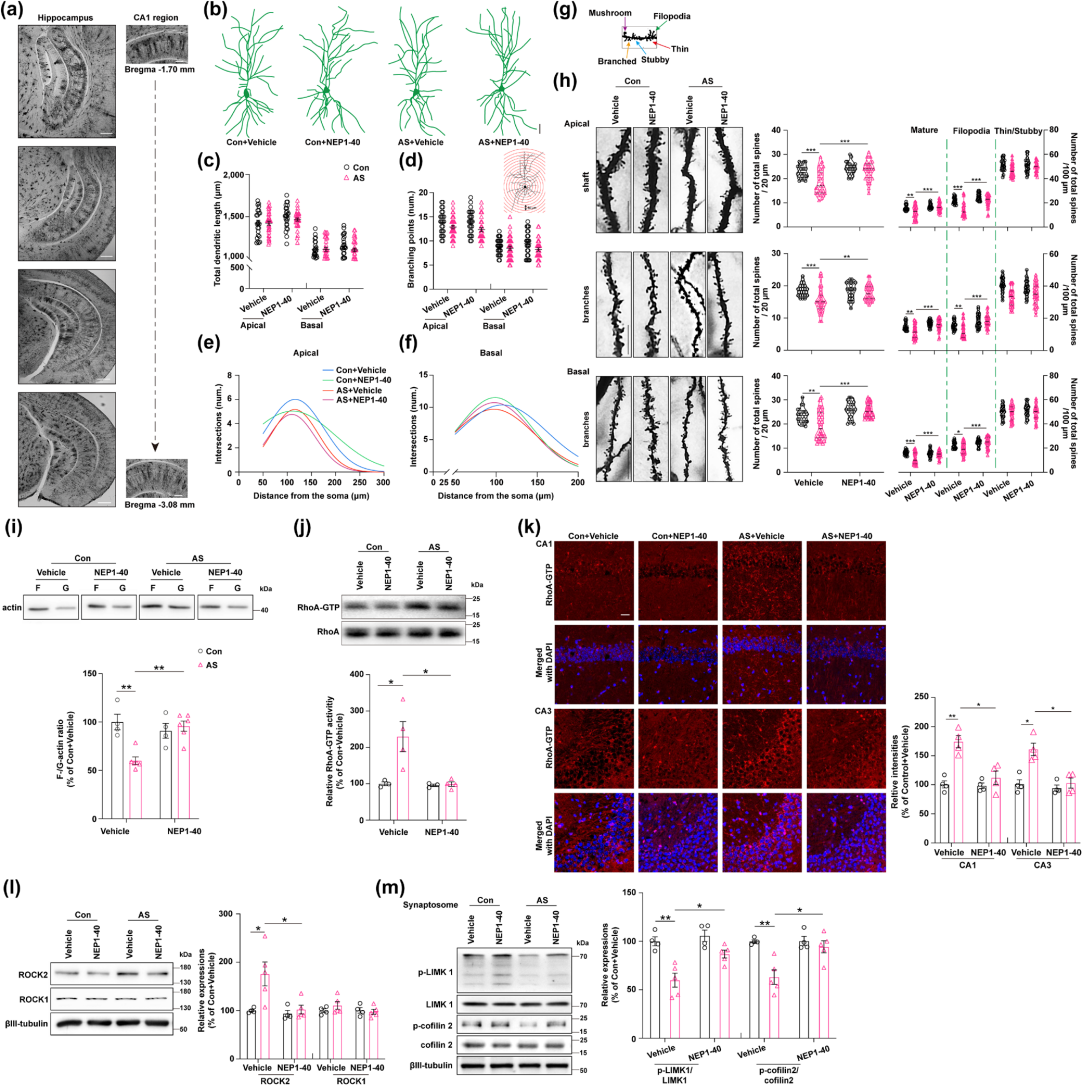
Dysregulation of hippocampal dendritic spine remodeling in aged mice following exposure to anesthesia and surgery: Involvement of F‐/G‐Actin balance, RhoA enzyme activity, and Nogo66‐NgR1 signaling. (a) Coronal hippocampus slices ranging from −1.70 mm to −3.08 mm relative to bregma were obtained by stitching together images captured at 10× magnification. Additionally, representative regions of the hippocampal CA1 (−1.70 mm and −3.08 mm relative to bregma) were imaged at 40× magnification. (b) The representative Golgi staining was performed to visualize the dendritic morphology in CA1 pyramidal neurons. Scale bar = 50 μm. (c–f) A schematic representation of Sholl's analysis method was used to assess the dendritic branching in hippocampal pyramidal neurons. Successive radial segments of 25 μm were measured from the center of the soma, which served as the reference point at the center of the circle. The Fiji software, along with NeuronJ and Sholl's analysis plug‐ins, was utilized to calculate the total dendritic length, number of branching points, and intersection number in both apical and basal dendrites for qualification qualitative analysis. (g) The classification of spines was visually represented, with thin, filopodia, stubby, mushroom, and branched spines indicated by red, green, blue, purple, and orange arrows, respectively. It is important to note that mushroom and branched spines are recognized as mature synapses. (h) On the left side, a representative spine on the shaft, apical, and basal dendrites in the hippocampal CA1 region was displayed. Scale bar = 10 μm. The number of spines/20 μm, mature/100 μm, filopodia spines/100 μm, and thin/stubby spines/100 μm were all quantified in the shaft, as well as the branches of apical or basal dendrites, across the four groups. (a–h) *N* = 24 neurons (8 neurons/mice) of the Con + Vehicle and Con + NEP1‐40 groups, *n* = 32 neurons (8 neurons/mice) of the AS + Vehicle and AS + NEP1‐40 groups, respectively. (i) The expressions of F‐actin and G‐actin in the hippocampus were examined using WB, and the ratio of F‐/G‐actin was analyzed across the four groups (*n* = 4 of the Con + Vehicle and Con + NEP1‐40 groups, *n* = 6 of the AS + Vehicle and AS + NEP1‐40 groups, respectively). (j) The relative activity of hippocampal RhoA was measured in the four groups (*n* = 3 of the Con + Vehicle and Con + NEP1‐40 groups, *n* = 4 of the AS + Vehicle and AS + NEP1‐40 groups, respectively). (k) The expression of RhoA‐GTPase in hippocampal CA1 and CA3 subregions was examined through IF staining (*n* = 4/group). Scale bar = 50 μm. (l) The expressions of the hippocampal ROCK1 and ROCK2, which are downstream of RhoA‐GTPase, were assessed using WB in the four groups. (m) The relative expressions of pSer3‐cofilin 2/cofilin 2 and p‐LIMK1/LIMK1, which are involved in F‐actin polymerization and regulated by RhoA‐GTPase activity, were analyzed in hippocampal synaptosomes across the four groups. (l–,m) *N* = 4 of the Con + Vehicle and Con + NEP1‐40 groups, and *n* = 5 of the AS + Vehicle and AS + NEP1‐40 groups, respectively. All data were analyzed by 2‐way ANOVA (2 × 2 factors: Groups × treatments) followed by Turkey's post hoc test and presented as mean ± SEM. *p < 0.05, **p < 0.01, and ***p < 0.001.

Given the high enrichment of NgR1 in synaptic fractions (Wills et al., 2012; Zhao et al., 2017), understanding how its ligand binding affects downstream signaling is key to elucidating its role in dendritic spine remodeling in aged mice after anesthesia and surgery. Representative coronal hippocampal slices, ranging from bregma −1.70 mm to −3.08 mm, were selected to examine changes in the CA1 pyramidal neurons (Figure 4a). We noted no significant differences in total dendritic length, number of branching points, or intersection numbers in apical and basal dendrites among the four groups (Figure 4b–f). This indicates that neither anesthesia and surgery nor NEP1‐40 treatment significantly altered the morphologies of CA1 pyramidal neurons. The quantitative examination of dendritic spine remodeling is essential for comprehending the mechanisms behind structural neuronal plasticity and the underlying causes of brain pathology. We subsequently analyzed the distribution of dendritic spines of CA1 pyramidal neurons with different types, including mature (mushroom and branched), filopodia, thin, and stubby (Figure 4g). We found that the number of the total, mature, and filopodia spines on apical and basal dendrites was reduced in the anesthesia and surgery + vehicle group compared to the control + vehicle group, which was reversed by NEP1‐40 treatment as compared to the anesthesia and surgery + vehicle group (Figure 4h). However, the number of thin/stubby spines per 100 μm on the apical and basal dendrites did not differ among the four groups (Figure 4h). Anesthesia and surgery impacted the distribution of dendritic spines, particularly reducing the number of the total, mature, and filopodia spines. Conversely, NEP1‐40 treatment counteracted this reduction, suggesting a potential role for NgR1 in modulating dendritic spine remodeling in aged mice after anesthesia and surgery.

Dendritic spines display a significant concentration of F‐actin, wherein multiple F‐actin binding proteins play crucial roles in regulating their shape and abundance. The actin cytoskeleton within these spines undergoes rapid and long‐lasting reorganization during events related to long‐term plasticity (Kommaddi et al., 2018). The induction of LTP triggers a shift in the G‐/F‐actin ratio, promoting the assembly of F‐actin in response to activity‐driven spine growth. The destabilization of the actin cytoskeleton via RhoA signaling leads to the collapse of growth cones (Li et al., 2023). Considering that NgR1 signaling has been demonstrated its ability to regulate the synaptic turnover (Akbik et al., 2013), we hypothesize that NgR1 located at synaptic sites regulates the levels of RhoA activity, thereby influencing the assembly of F‐actin and further impacting the spine morphogenesis or dynamic changes. In our study, we first detected the changes in hippocampal F‐/G‐actin ratio after the behavior tests. The F‐/G‐actin ratio in the hippocampus was diminished in the anesthesia and surgery + vehicle group compared to the control + vehicle group but was restored following NEP1‐40 treatment in comparison to the anesthesia and surgery + vehicle group (Figure 4i), suggesting that the hippocampal F‐actin assembly was disturbed in the aged mice after being exposed to anesthesia and surgery. To further investigate the disruption of actin dynamics induced by anesthesia and surgery in aged mice, we simulated this stress in rat hippocampal neuronal cultures by coculturing them with LPS‐pretreated BV2 microglial cells. We used phalloidin staining to label F‐actin and found that the number of filopodia‐like clusters was decreased while spine‐like structures were increased from DIV4 to 16 (Figure S2a). Therefore, we chose to treat neurons after at least DIV15‐16 in culture for the in vitro studies (Figure S2b). In comparison to the control group cocultured with vehicle‐treated BV2 microglia (the vehicle group), coculturing with LPS‐pretreated BV2 microglia led to increased expressions of NgR1, NogoA, and NgR1 coreceptors including LINGO1, TROY, and P75NTR in the hippocampal neurons (the LPS + vehicle group), and Nogo66 inhibitory peptide Nogo‐P4 (residues 31–55 of Nogo‐66), an active segment of Nogo‐66, and a potent inhibitory component of Nogo‐A (GrandPre et al., 2000) induced similar increases of these compared to the vehicle group (Figure S2c). Immunocytochemistry staining of NgR1 showed comparable alterations (Figure S2d). Compared to the control + vehicle group, a decrease of phalloidin‐labeled F‐actin expression was observed in the LPS + vehicle group, and this decrease was replicated by Nogo‐P4 treatment, indicating a reduced F‐actin; this reduction was reversed with NEP1‐40 and F‐actin stabilizer Jasplakinolide (Jasp) treatments (Figure S2e).

Subsequently, we detected the activity of RhoA‐GTPase, relatively represented by the ratio of RhoA‐GTPase to Total‐RhoA, was increased in the anesthesia and surgery + vehicle group relative to the control + vehicle group, which was normalized upon NEP1‐40 treatment compared to the anesthesia and surgery + vehicle group (Figure 4j). Immunofluorescence staining of RhoA‐GTPases showed comparable alterations (Figure 4k). We also investigated the proteins downstream of RhoA‐GTPases that regulate F‐/G‐actin equilibrium. ROCK2 rather than ROCK1 expression, both as the downstream of the RhoA pathway, was upregulated in the anesthesia and surgery + vehicle group compared to the control + vehicle group, and this upregulation was reversed by NEP1‐40 treatment (Figure 4l). Furthermore, the key enzymes involved in F‐/G‐actin equilibrium, including p‐LIMK1/LIMK1 and pSer3‐cofilin 2/cofilin 2 in the hippocampal synaptosome were reduced in the anesthesia and surgery + vehicle group compared to the control + vehicle group but were increased with NEP1‐40 treatment (Figure 4m). Moreover, the application of Fasudil, a potent inhibitor of the RhoA‐ROCK pathway, did not result in any observable differences in the behavioral tests between the control + fasudil and the anesthesia and surgery + fasudil groups (Figure S3A–I).

### 3.5 Restoring experience‐driven AMPA receptors delivery to synapses and enhancing neuronal activity in the hippocampus in anesthesia and surgery‐exposed aged mice through NEP1‐40 treatment

**FIGURE 5:**
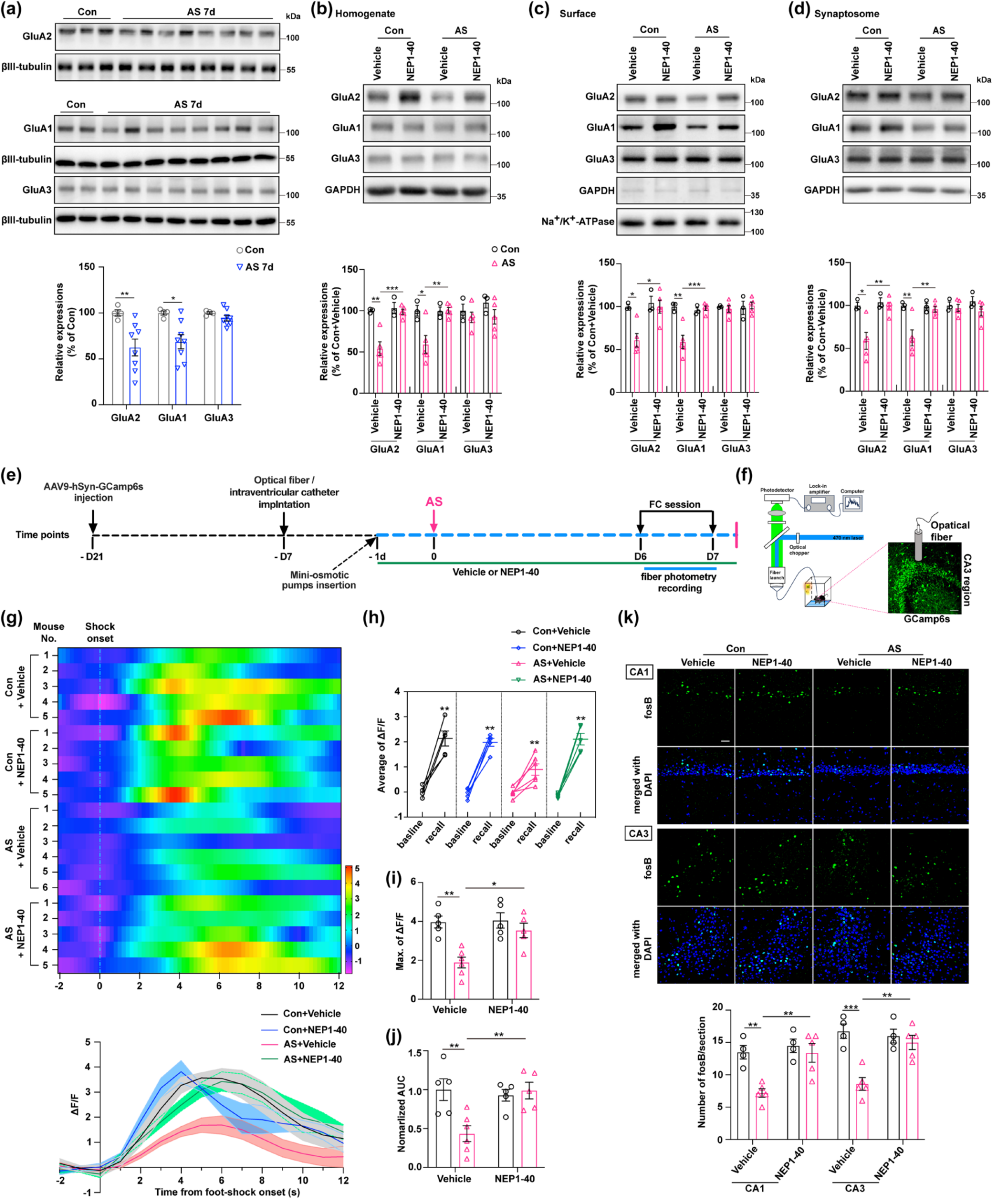
Effects of AS and NgR1 antagonistic Peptide NEP1‐40 on synaptic AMPAR delivery and neuronal activity in the hippocampus of aged mice. (a) The expression levels of AMPA receptors (GluA1, GluA2, and GluA3) in the hippocampus were quantified in both the Con and AS 7 d groups (*n* = 4 of the Con group, *n* = 8 of the AS 7 d group). (b) The quantification of GluA1, GluA2, and GluA3 expressions in the hippocampal homogenate was performed following the administration of AS, Con, Vehicle, and NEP1‐40. (c) The quantification of GluA1, GluA2, and GluA3 expressions on the hippocampal membrane (surface) was performed after administering AS, Con, Vehicle, and NEP1‐40 treatments. (d) The assessment of GluA1, GluA2, and GluA3 expression in hippocampal synaptosomes was conducted following the administration of Con, AS, Vehicle, and NEP1‐40. (b–d) *N* = 3 of the Con + Vehicle and Con + NEP1‐40 groups, and *n* = 5 of the group AS + Vehicle and AS + NEP1‐40 groups. (e) The diagram illustrated the application of fiber photometry for calcium imaging in aged mice during the FC test, with interventions including Con, AS, Vehicle, and NEP1‐40. (f) The schematic diagram illustrating Ca^2+^ signal recording using fiber photometry in the CA3 subregion of the hippocampus. Expression of the AVV‐hSyn‐GCaMP6s virus in the CA3 region at a 40× magnification. Scale bare = 50 μm. (g) Heatmap illustrating changes in Ca^2+^ signals during the process of freezing in the contextual FC test among the four groups. ΔF/F represented a deviation in the fluorescence of GCaMP6 from the baseline. (h) The average ΔF/F fluorescence of GCaMP6 was measured between the baseline and recall (freezing) periods during the contextual FC test, comparing across the four groups. (i) The maximum ΔF/F of GCaMP6 fluorescence during the freezing behavior session in the contextual FC text was compared across the four groups. (j) The area under the curve (AUC) of the average ΔF/F data represents the normalized fluorescence of GCaMP6 during the freezing behavior session among the four groups. (g–j) *N* = 5 of the Con + Vehicle, Con + NEP1‐40, and AS + NEP1‐40 groups; *n* = 6 of the AS + Vehicle group. (k) The expression of fosB in the CA1 and CA3 subregions of the hippocampus was evaluated through IF staining. Quantification of fosB‐positive cells per slice in the CA1 and CA3 subregions of aged mice was conducted at a 40× magnification after administering various treatments, including Con, AS, Vehicle, and NEP1‐40 (*n* = 4 of the Con + Vehicle and Con + NEP1‐40 groups, *n* = 5 of the AS + Vehicle and AS + NEP1‐40 groups, respectively). Scale bare = 50 μm. (a, h) Data were analyzed by an unpaired *t* test with the Mann–Whitney *U* test; (b–d, g, i–k) All data were analyzed by 2‐way ANOVA (2 × 2 factors: Groups × treatments) followed by Turkey's post hoc test and presented as mean ± SEM. *p < 0.05, **p < 0.01, and ***p < 0.001.

Previous studies have provided evidence of the dynamic trafficking of AMPARs playing a crucial role in synaptic remodeling and precisely regulating the number and subtype of AMPARs at the synapse is essential for controlling excitatory neurotransmission, synaptic plasticity, and the formation of neural circuits that facilitate normal neurocognitive function (Cingolani & Goda, 2008). However, a comprehensive understanding of the spatial and temporal regulation of actin dynamics with AMPARs and the postsynaptic density (PSD) remains necessary. To investigate the alterations in APMARs delivery to synapses, we analyzed the expressions of GluA1, GluA2, and GluA3—which are predominantly enriched in the pyramidal neurons and primarily form GluA1/2 and GluA2/3 heterotetrameric types—in hippocampal homogenate, membrane fractions (surface), and synaptosomes after the FC test. We observed that the expressions of GluA1 and GluA2, but not GluA3, in hippocampal homogenate were reduced at 7 d after AS compared to the control group (Figure 5a). Similarly, the expressions of GluA1 and GluA2, but not GluA3, in the hippocampal homogenate, surface, and synaptosomes were diminished in the anesthesia and surgery + vehicle group compared with the control + vehicle group (Figure 5b–d). Notably, NEP1‐40 treatment ameliorated this reduction as opposed to the anesthesia and surgery group (Figure 5b–d).

**FIGURE 6:**
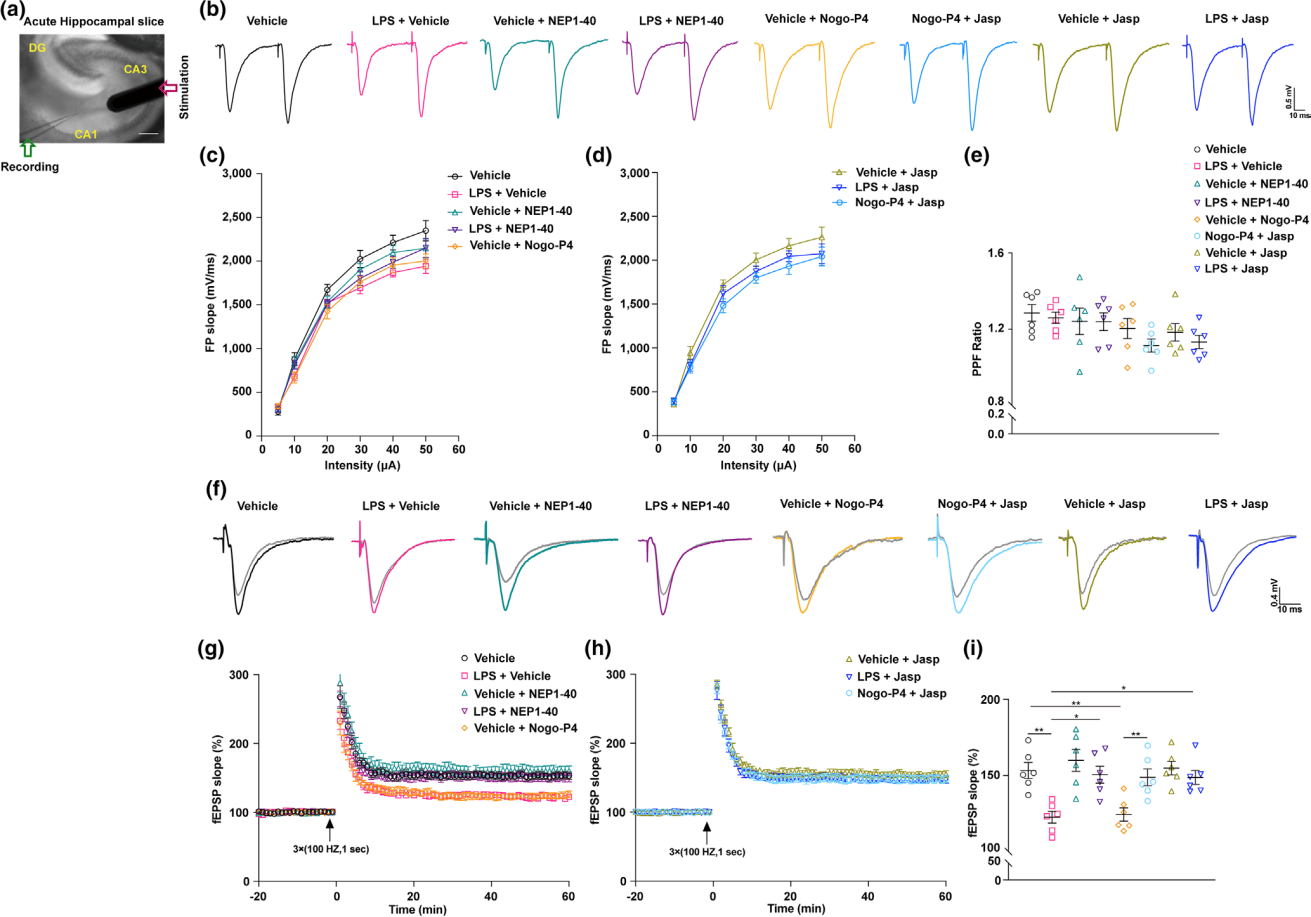
Effects of LPS, Nogo‐P4, NEP1‐40, and F‐actin stabilizer Jasplakinolide on LTP at the Schaffer collateral‐CA1 pathway. (a) The diagram depicts the process of LTP recording in acute hippocampal slices from adult mice, specifically within the Schaffer collateral‐CA1 pathway. It showcases both a stimulation electrode and a recording electrode. The illustration further highlights the hippocampal subregions, namely CA1, CA3, and DG. Scale bar = 100 μm. (b–e) Paired pulse facilitation (PPF) recordings were conducted in adult mice following intracerebroventricular (ICV) injection of PBS or LPS, with subsequent administration of NEP1‐40, Nogo‐P4, or Jasp. The recordings included an analysis of the field potential (FP) slope and PPF ratio. (f–h) LTP recordings of fEPSP were conducted in acute hippocampal slices obtained from adult mice (8 weeks old). These recordings were performed 6 h after intracerebroventricular (i.c.v) injection of either saline or LPS. Following poststimulation with three consecutive trains at 100 Hz for 1 s (indicated by a black arrow), the effects of NEP1‐40, Nogo‐P4 peptide, or Jasp applications were evaluated. (i) The percentage of mean fEPSP at 51–60 min after the application of three trains of stimulation (100 Hz, 1 s each) was analyzed. This analysis was performed on adult mice that were pretreated with either PBS or LPS and subsequently received NEP1‐40, Nogo‐P4 peptide, or Jasp applications. Data were analyzed by one‐way ANOVA followed by Turkey's test and presented as mean ± SEM. *N* = 6 slices (2 slices/mice) of each group. *p < 0.05 and **p < 0.01.

To further detect the effects of dysfunction of actin dynamic induced by Nogo66‐NgR1 signaling on synaptic plasticity and strength, we examined the total expressions of PSD95, GluA1, and GluA2 in the cultured hippocampal pyramidal neurons. We noticed that the coculture of LPS pretreated BV2 microglia induced decreased expressions of s‐GluA1, PSD95, and s‐GluA1/PSD95 colocalization on the hippocampal neuron cultures, which was mimicked by Nogo‐P4 intervention compared with the Vehicle group (Figure S4). The applications of NEP1‐40 and Jasp caused an increase in the levels of s‐GluRA1, PSD95, and s‐GluA1/PSD95 colocalization (Figure S4). Similarly, the expressions of s‐GluA2, PSD95, and s‐GluA2/PSD95 colocalization were decreased in the hippocampal neuron cultures after being cocultured with the LPS pretreated microglia cells, which was mimicked by Nogo‐P4 intervention compared with the vehicle group (Figure S5). The applications of NEP1‐40 and Jasp caused an increase in the levels of s‐GluA2, PSD95, and s‐GluA2/PSD95 colocalization (Figure S5). In the mammalian brain, AMPARs play a vital role in facilitating fast excitatory transmission, and their modulation is instrumental in fine‐tuning synaptic efficiency during activity‐dependent plasticity. Hence, unraveling the mechanisms by which synaptic molecules, specifically AMPARs, facilitate neuronal communication and monitor their dynamic expression throughout different behaviors is imperative. This understanding holds great significance in comprehending neurocognition as well as various diseases affecting the brain. The procedures and results for stimulating and recording PPF and LTP of excitatory neurotransmission in hippocampal slices are depicted in Figure 6a. We observed no significant differences in PPF ratio and FP slope, regardless of LPS exposure, Nogo‐P4 intervention, or NEP1‐40 and Jasp application compared to the vehicle group (Figure 6b–e). This indicates that presynaptic transmission was unaffected and remained intact. Subsequently, the fEPSP of hippocampal slices was recorded. It was noted that LPS‐induced brain infection led to a decrease in the fEPSP slope, an effect that was replicated by the Nogo‐P4 intervention; conversely, treatment with NEP1‐40 and Jasp increased the fEPSP slope (Figure 6f–i), suggesting that LTP impairments were attributable to postsynaptic damage. These data were also consistent with that shown in Figure 3.

To gain a deeper understanding of the effects of NEP1‐40 treatment on neuronal activity and excitatory transmission after reintroducing AMPARs delivery to synapses in the aged mice following anesthesia and surgery exposure, we performed calcium imaging using fiber photometry during the sessions of fear conditioning test (Figure 5e). The mice were subjected to stereotaxic injections of an AAV9 vector carrying the calcium indicator GCamp6s specifically in the CA3 region. Additionally, an optical fiber was surgically implanted above the CA3 region to monitor and record cellular activity (Figure 5f). Some mice underwent one‐time or two‐time surgical procedures for osmotic minipump and optical fiber implantation before anesthesia and laparotomy surgery which may impact the expression of NgR1 in the hippocampus. However, no significant difference in the hippocampal NgR1 expression was found among the control without any surgery, one‐time surgery, and two‐time surgeries in aged mice (Figure S6). In comparison to the control + vehicle group, the neurons in the CA3 region of aged mice exposed to anesthesia and surgery treated with vehicle displayed a consistent decay in calcium responses during the contextual FC test. This decline indicated a reduction in neuronal activity caused by anesthesia and surgery exposure. However, when treated with NEP1‐40, the decay in calcium responses was reversed, suggesting a potential therapeutic effect of NEP1‐40 on restoring neuronal activity in this context (Figure 5g). Both the average and maximum values of △F/F, as well as the normalized area under the curve (AUC), exhibited a decrease in the anesthesia and surgery + vehicle group relative to the control + vehicle group. However, this decline was mitigated by the administration of NEP1‐40, indicating its potential to alleviate the observed reductions in △F/F and normalized AUC in the anesthesia and surgery‐exposed mice (Figure 5h–j). Additionally, the immunofluorescence staining of fosB exhibited similar changes in the CA1 and CA3 regions among all four groups (Figure 5k). These data provide compelling evidence that in aged mice exposed to anesthesia and surgery, the increased hippocampal Nogo66‐NgR1 signaling inhibits the efficient delivery of AMPARs to synapses, resulting in reduced excitatory transmission and neuronal activity. This impairment predominantly arises from the inhibition of F‐actin disassembly. Encouragingly, treatment with NEP1‐40 proves successful in ameliorating these impairments and restoring proper AMPARs delivery to synapses.

## 4 DISCUSSION

**FIGURE 7:**
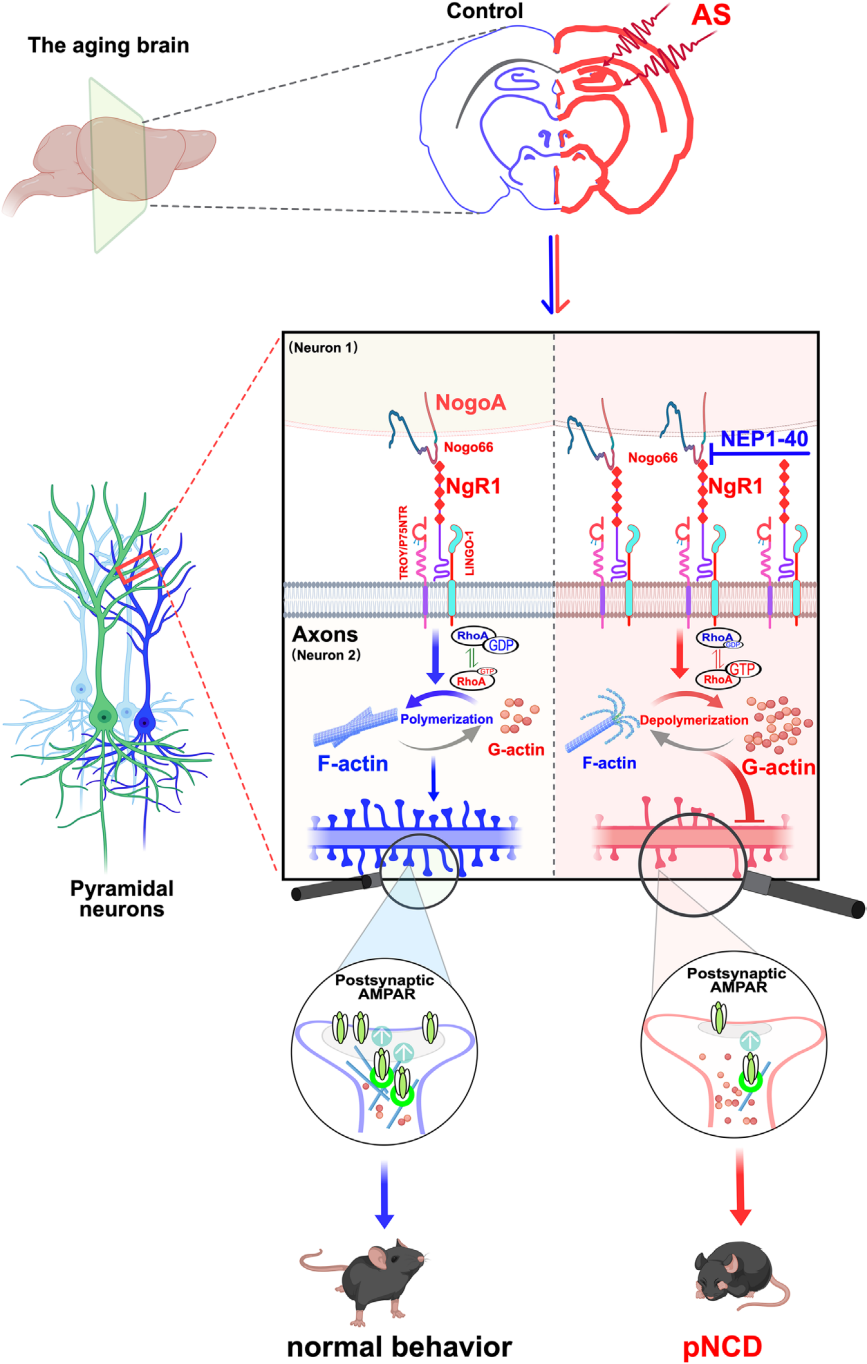
The potential molecular mechanism of Nogo66‐NgR1 signaling negatively regulates neurocognitive disorders in aged mice following anesthesia and surgery (AS). The cartoon illustrates the alterations in intracellular pathways associated with actin dynamics, synaptic remodeling, and synaptic delivery of AMPARs mediated by Nogo66‐NgR1 signaling in the hippocampus. In aged mice following AS, these changes are reversed by treatment with the NgR1 competitive antagonist NEP1‐40. In the hippocampus of aged mice, AS exacerbated the aging‐induced stress, resulting in the sustained overexpression of hippocampal NgR1 and NogoA. NgR1 progressively binds to its transmembrane transduction partners (LINGO‐1/TROY/P75NTR), leading to actin filament depolymerization (F‐/G‐actin ratio turnover or F‐actin disequilibrium) and upregulation of downstream RhoA‐GTPase activity. This dysregulation hampered the surface delivery of synaptic GluA1 and GluA2 subunits of AMPARs, impairing synaptic metaplasticity after experiences and ultimately causing anxiety‐like behavior and deficits in hippocampus‐dependent contextual fear memory. AS, anesthesia and laparotomy surgery; F‐actin, filamentous actin; G‐actin, globular actin; LINGO1, leucine‐rich repeat and immunoglobulin domain‐containing 1; NgR1, Nogo66 receptor 1; NogoA, neurite outgrowth inhibition protein A; P75NTR, neurotrophin receptor P75; pNCD, postoperative neurocognitive disorders; RhoA‐GTP, small‐GTPases; TROY, orphan receptor of tumor necrosis factor‐α receptor superfamily member 19.

Our study found that in aged mice exposed to anesthesia and surgery, hippocampal Nogo66‐NgR1 signaling was excessively activated particularly in the CA1 and CA3 subregions. Concurrently, this heightened NgR1 signaling adversely affected synaptic plasticity which was primarily due to the dysregulation of F‐/G‐actin equilibrium, leading to disordered spine types and disrupted postsynaptic AMPARs delivery. This disruption was mediated through the upregulated activity of downstream molecule‐RhoA‐GTPases, culminating in anxiety‐like behavior and impaired hippocampus‐dependent contextual fear memory. Intracerebroventricular injection of NEP1‐40, the Nogo‐A extracellular peptide comprising the first 40 amino acids of Nogo‐66, by competitively binding to NgR1, attenuated NgR1 signaling and halted the downstream cascade, reinstated actin dynamics and postsynaptic AMPARs delivery, and ameliorated anxiety‐like behavior and hippocampus‐dependent contextual fear memory impairments. Additionally, the Rho kinase inhibitor Fasudil was proved to be therapeutically effective under the current study settings. Thus, our findings suggest that targeting hippocampal Nogo66‐NgR1 signaling may offer new insights into the mechanisms of pNCD and serve as a promising therapeutic strategy to be developed to tackle pNCD (Figure 7).

Numerous studies underscored the importance of establishing an appropriate number of synaptic connections during experience processes for optimal brain function (Holtmaat & Svoboda, 2009; Rabinowitch et al., 2024). Excitatory synaptic remodeling initiates with the formation of connections between axons and dendritic filopodia, which are actin‐rich protrusions along dendritic shafts. Synaptic strength alterations are central to experience, with long‐lasting changes involving variations in the number and size of dendritic spines (Connor & Siddiqui, 2023; Dalva et al., 2007). These processes include regulating synaptogenesis onset and location, restricting synaptic components recruitment to inappropriate sites, limiting synapse growth, or pruning weak synaptic contacts during synapse elimination. In adults, anatomical plasticity is more restricted, and synaptic connectivity changes are implicated in neurocognitive tasks, where synaptic plasticity fine‐tunes neuronal networks during experience (Brosens et al., 2023; Sheng et al., 2018). Failures in this critical process are believed to contribute to neurological disorders including depression, autism, and AD (Brown & Gould, 2024; Morimura et al., 2017; Pinky et al., 2023). While numerous studies in the field of pNCD have identified several cell–cell recognition molecules that promote synapse formation, there is comparatively less understanding of the mechanisms that limit synapse numbers. This regulation is crucial to maintain the precise organization of neural circuits, particularly in brains affected by pNCD.

The Nogo receptor (NgR) family serves as a pivotal regulator, limiting the assembly, and plasticity of synapses. Amongst these receptors, NgR1 plays a significant role in determining the set point for experience‐dependent synaptic turnover. It exerts control over circuit remodeling and synaptic strength, while also uniquely influencing the dynamics of dendritic spines and metaplasticity in adult neurons (Akbik et al., 2013). Loss of the NgR1 increases excitatory synapse number in the hippocampus, triggered by new synapse assembly resulting from NgR loss on dendrites (Wills et al., 2012; Zhao et al., 2017). This occurrence stems from the promotion of new synapse formation due to the absence of NgR‐mediated inhibition. Additionally, NgR1 acts as a receptor for Aβ, which inhibits synapse assembly and potentially initiates cognitive pathology in Alzheimer's disease (Zhao et al., 2017). Consequently, NgR1 is hypothesized to serve as a regulatory brake on the anatomical dynamics associated with experience‐related neuronal activity. Therefore, understanding the critical role of NgR1 in governing synaptic remodeling during experience becomes crucial in exploring the development of age‐related cognitive decline in mice. Our research findings demonstrate that both NgR1 and its key ligand, NogoA, are consistently upregulated in the hippocampus following anesthesia and surgery, particularly in the CA1 and CA3 subregions. This upregulation persists for up to 7 days after anesthesia and surgery and coincides with anxiety‐like behavior and impairments in hippocampus‐dependent contextual fear memory. Subsequently, we delved deeper into investigating the mechanisms by which NgR1 signaling influences the brain in a state of pathological neurocognitive decline.

Dendritic spines, serving as postsynaptic receptive regions for most excitatory synapses, play a pivotal role in higher brain functions like emotion and memory through their morphological plasticity. In this study, we found that there was a noticeable reduction in the number and types of spines, predominantly mature and filopodia in the CA1 region, while neuron morphology remained unaffected after anesthesia and surgery in aged mice. Given that NgR1 is expressed primarily in the dendritic shaft where it colocalizes with filamentous actin (Wills et al., 2012); actin microfilaments, particularly concentrated in dendritic spines and growth cones, play a vital role in synaptic remodeling. The polymerization and depolymerization of F‐actin are crucial for dendritic spine morphology and synaptic‐activity‐dependent structural alterations (Ripoli et al., 2023). Given the high concentration of F‐actin in dendritic spines, maintaining the F‐/G‐actin ratio is vital for optimal synaptic functional integrity. This change in actin dynamics is regulated by molecules like small GTPases including the Rho family (Stern et al., 2021). Previous studies showed that NgR1 binding to Nogo‐A affects CNS axonal regeneration by activating Rho‐GTPase signal pathways, suggesting that targeting NgR1 may promote recovery from CNS injuries (Schwab, 2010; Xiao et al., 2022). In our study, we observed that the upregulated hippocampal Nogo66‐NgR1 signaling following anesthesia and surgery activated the RhoA‐GTPase, leading to F‐actin depolymerization and neurocognitive disorders in aged mice. Moreover, NEP1‐40 treatment can reverse these changes. The findings of this study demonstrated that hippocampal Nogo66‐NgR1 signaling acts as a negative regulator in the development of postoperative neurocognitive disorders in aged mice.

Previous studies illustrated that neuronal activity regulates synaptic AMPARs trafficking, which is essential for normal neurocognitive function and implicated in various brain diseases (Yang et al., 2018). Interestingly, our data were a mirror of the previous study, showing that a loss of NgR1 increased dendritic spine numbers, PSD95, and AMPA receptor subunit GluA2 (Wills et al., 2012). We found that the upregulated hippocampal Nogo66‐NgR1 signaling was mainly induced by postsynaptic damage, and synaptic GluA1 and GluA2 in the hippocampus were decreased and concomitant with decreased neuronal activity during behavioral tests in aged mice exposed to anesthesia and surgery, which were prevented by treatment with NEP1‐40. Our findings indicate that upregulated hippocampal Nogo66‐NgR1 signaling disrupts F‐/G‐actin equilibrium via activating RhoA‐GTPase, which leads to restrained postsynaptic AMPARs delivery, culminating in anxiety‐like behavior, and hippocampus‐dependent contextual fear memory impairments in aged mice after anesthesia and surgery.

Potential mechanisms involved in the increase of hippocampal NgR1 following anesthesia and surgery remains unknown. These may be due to inflammatory responses which were well documented as they increased the NgR1 expression (Fry et al., 2007; Theotokis et al., 2016). Another mechanism may be related to matrix metallopeptidase MT3‐MMP (named MMP16) that promoted synaptic NgR1 ectodomain shedding and subsequently enhanced excitatory synapse formation (Sanz et al., 2018). Indeed, the expression of MT3‐MMP was decreased after anesthesia reported previously (Zhang et al., 2019), and, therefore, NgR1 was likely increased due to its insufficient shedding due to the decrease in MT3‐MMP. There are also some other reasons associated with the changed NgR1 expression after anesthesia and surgery. For example, specific endogenous NgR1 antagonists, such as CRTAC1/LOTUS and LGI1, which are known to inhibit NgR1 pathway signaling (Sato et al., 2011; VanGuilder Starkey et al., 2013). It was found that these endogenous inhibitors of the NgR1 pathway decreased significantly with aging and cognitive decline, with lower expression levels correlating with diminishing cognitive abilities, particularly in the hippocampus (VanGuilder Starkey et al., 2013). LGI1, specifically binding to NgR1 but not its structural homologs NgR2 or NgR3, interacts at a site overlapping the Nogo66 binding region through an alternate co‐receptor, ADAM22. Disruption in the LGI1‐ADAM22 synaptic complex has been linked to abnormal AMPARs‐mediated synaptic transmission and epilepsy (Fukata et al., 2006). However, in our study, we mainly investigated the expressions of NogoA and NgR1‐associated signaling but did not detect the changes in endogenous NgR1 antagonist expressions. Additionally, although our study focused on the mechanisms in excitatory neurons, it is crucial to note that NgR1 is also expressed in inhibitory interneurons (Bhagat et al., 2016). Previous studies have highlighted the role of NgR1 in limiting disinhibition microcircuit involvement, particularly in parvalbumin‐expressing interneurons, which are pivotal in closing the critical period for ocular dominance plasticity (Stephany et al., 2016). Thus, future research should aim to explore and improve our understanding of NgR1's effects on inhibitory interneurons in the context of pNCD and continue to deeply explore the above possible mechanisms.

Our work has some limitations. Firstly, we applied LPS in our in vitro experiments to mimic systemic inflammation following anesthesia and surgery although it is often used in in vitro (Chen et al., 2019; Kong et al., 2024; Wang et al., 2023) and in vivo models of various diseases/conditions (Batista et al., 2019; Chen et al., 2019; Dias‐Carvalho et al., 2024). However, how this setting represents in animals following anesthesia/surgery is unknown and warrants further study. Secondly, mice were used in vivo part but neuron‐BV2 glia cocultures derived from rats were used for the in vitro study to verify the underlying mechanisms of Nogo66‐NgR1 signaling, which is similar to the previous study using rat neurons to verify the mechanisms found in mice (Wills et al., 2012). Thirdly, only male‐aged mice were used in this study. However, male versus female‐aged mice are different in certain aspects including reproductive senescence and physiology (Diaz Brinton, 2012; Levy et al., 2023). Therefore, aged female subjects are needed in future studies to enhance gender generality and underlying mechanisms of disease conditions reported here.

In conclusion, our study indicates that heightened Nogo66‐NgR1 signaling in the hippocampus may play a detrimental role in anxiety‐like behavior and hippocampus‐dependent contextual fear memory in pNCD mice. This negative impact involves a disrupted F‐/G‐actin equilibrium and reduced activity‐dependent surface delivery of postsynaptic AMPARs following behavioral tests. Notably, administration of the NgR1 neutralizing peptide antagonist NEP1‐40 or the Rho kinase inhibitor Fasudil effectively mitigated these impairments. This suggests that hippocampal Nogo66‐NgR1 signaling may be a key player in excitatory transmission in age‐related neurocognitive disorders after anesthesia and surgery.

## AUTHOR CONTRIBUTIONS

JM conducted the anesthesia and laparotomy surgery, IF/ICC, cannula placement, in vivo fiber photometry, synaptosome and membrane protein extraction, and BV2/neuronal culture. JM, LGZ, and CJ carried out the behavioral test, WB, CO‐IP, Nissl staining, F‐/G‐actin in vivo assay, RhoA pull‐down activation assay, and analyzed the data. JM and LGZ drafted the original manuscript. CJ did the acute hippocampal slice for electrophysiology. TXH did the Glogi staining and the tissue embedding. ZYY took care of the animals in this study. ZYY and YGL did qRT‐PCR, drug treatment. SSY, MDQ, JMH, and YJJ supervised the project and edited and finalized the manuscript. All co‐authors agreed and approved the submission.

## CONFLICT OF INTEREST STATEMENT

The authors declare no competing interests.

## Supporting information


Appendix S1.

## Data Availability

All raw data used in this manuscript are available on request.
