# Supplementary material for: Hippocampal Nogo66‐NgR1 signaling activation restricts postsynaptic assembly in aged mice with postoperative neurocognitive disorders
Source: Aging Cell. 2024 Oct 16;24(1):e14366. doi: 10.1111/acel.14366 (PMC11709113; doi:10.1111/acel.14366)
Supplement: Supplementary file 1 — Appendix S1. [file ACEL-24-e14366-s001.zip › Supplement methods and figures.docx]

**Online supplemental Figures for Jia M, et al.** **Hippocampal Nogo66-NgR1 signaling activation restricts postsynaptic assembly in aged mice with postoperative neurocognitive disorders**

**Online supplemental methods for Jia M, et al. Hippocampal Nogo66-NgR1 signaling activation disrupting synapse assembly and mediating postoperative neurocognitive disorders in aged mice**

**In vivo experiments**

***Laparotomy surgery***

The aged mice (20-22 M old) were randomly assigned to either the control group or the Anaesthesia and laparotomy surgery (AS) group. The AS procedure was conducted as previously described(Wei et al., 2023). The mice were initially anesthetized in a transparent acrylic chamber filled with 1.5% isoflurane monitored with an anesthetic gas monitor (Vamos, Draeger Medical AG & Co. KGaA, Germany) in 100% O_2_. During the aseptic abdominal exploratory surgery, a cone device was used to maintain isoflurane Anaesthesia close to the mouse's mouth throughout the procedure. A systematic examination of abdominal organs such as the liver, spleen, right kidney, bowel, and left kidney was performed for 10 min. Approximately 2.0 cm of the intestine was gently exteriorized and massaged for 30 seconds before being carefully repositioned within the peritoneal cavity while adhering to anatomical structures. Subsequently, the muscle wall and the skin were sutured with 5-0 and 4-0 sutures, respectively. Terramycin ointment was used to protect the eyes during surgery and Polysporin was applied to the external wound to prevent infection. Throughout the surgical process, a heating pad was utilized to maintain the rectal temperature at approximately 37°C. Control mice did not receive both Anaesthesia and surgery.

***Cannula placement***

Some cohorts of aged mice 24 h before AS described above were implanted with cannulas (BIC-3, H00304011, RWD Life Science Co., Ltd) under 1.5%-1.8% isoflurane anaesthesia. The cannula was inserted into the left lateral brain ventricle with the coordination of - 0.48 mm posterior and + 1.00 mm left relative to the bregma and - 2.25 mm deep using a stereotaxic apparatus (68025, RWD Life Science Co., Ltd). The osmotic minipump (1002W, RWD Life Science Co., Ltd) was pre-connected with a cannula via infusion catheter and then placed under the skin on the upper back. Other cohorts of aged mice 2 weeks before AS were implanted with a stainless-steel guide cannula (62003, O.D 0.48 mm × I.D 0.34 mm, RWD Life Science Co., Ltd) to left lateral ventricle under the same conditions as those for the subsequent fiber photometry recording. One day prior to AS, the cannula of the osmotic minipump was inserted into the stainless-steel guide cannula for lateral ventricle administration. All cannulas, guide cannulas, and ceramic fiber optic ferrules were fixed with screws (62513, RWD Life Science Co., Ltd, Shenzhen, China) by super-bond C&B dental adhesive resin cement (SUN MEDICAL CO., LTD, Japan) to the skull. The mice were kept on a heating pad set at 37°C throughout the procedure.

***Drug treatments***

To counteract the activation of NgR1 caused by the binding of the Nogo66 domain, we utilized an NgR1-neutralizing antagonist peptide known as NEP1-40. NEP1-40 competes with NgR1 and is composed of the initial 40 amino acids from the Nogo-66 segment of NogoA. Originally designed as a transactivator of transcription (TAT) for the human immunodeficiency virus-1 transduction system, the TAT-NEP1-40 peptide was synthesized in an acetate form and purified by MedChemExpress (MCE) Co., Ltd. located in Shanghai, China. For our experiments, we selected a concentration of 500 μM for TAT-NEP1-40 (HY-P5754), which was dissolved in 100 μl of a vehicle solution consisting of 98% PBS and 2% DMSO. The control vehicle solution contained the same mixture of PBS and DMSO(Fang et al., 2016; Rolando et al., 2012). To administer either NEP1-40 or the vehicle solution into the left ventricle, osmatic minipumps were applied and the administration period began 24 h before AS and lasted until the completion of the behavior test at a rate of 0.25 μL/min for 9 consecutive days. Mice were randomly assigned to one of four groups based on their treatment: control + Vehicle (Con + Vehicle), control + NEP1-40 (Con + NEP1-40), isoflurane Anaesthesia + laparotomy surgery + Vehicle (AS + Vehicle), and isoflurane Anaesthesia + laparotomy surgery + NEP1-40 (AS + NEP1-40).

To investigate the impact of RhoA-GTPase activity on the pathophysiology of pNCD in aged mice, we administered a targeted inhibitor of Rho-kinase called Fasudil (HY-10341A, MCE, Shanghai, China). The inhibitor was dissolved in normal saline at a concentration of 2 mg/mL and injected intraperitoneally at a dosage of 20 mg/kg per mouse(Bobo-Jimenez et al., 2017). The injections were given once a day starting from 1 h before AS and continued until the completion of the last behavioral test.

***Behavioral tests***

The behavioral experiments were carried out in sound-proofed rooms, adhering to the light cycle. The experiments followed a sequential order: marble burying, elevated zero-maze, open field, Y-maze, and fear conditioning tests. These tests took place between 2 to 7 days after AS. Meticulous observations of each mouse's behavior were made, either by an experimenter blinded to the experimental conditions or using a computer-operated video tracking system obtained from Shanghai Softmaze Information Technology Co. Ltd in Shanghai, China. To maintain cleanliness and eliminate any potential odor interference between trials, the chambers were thoroughly sanitized using MB-10 solution (100 ppm, CL-4136-15, CLEA Japan, Inc., Tokyo, Japan) or a 30% ethanol solution, followed by drying with paper towels.

1. *Marble burying test (MBT)*

On the morning (from 9:00 am to 11:00 am) of AS 2 d, the MBT was utilized to evaluate repetitive and anxiety-like emotionality and antianxiety activity in mice (de Brouwer et al., 2019). To experiment, prepare multiple mouse PC cages measuring 31 cm × 23 cm × 16 cm. Line the bottom of each cage with approximately 5 cm thick corn cob bedding. Introduce a mouse into each cage and allow them to acclimate for 15 min. Next, place 20 glass marbles with a diameter of approximately 16 mm on the bottom of each cage. Arrange the marbles in 5 rows, with 4 marbles in each row, spaced 3 cm apart in a regular pattern. Use a camera to record the behavior of the experimental mice as they bury the glass marbles within a 30-minute timeframe. After completing the experiment, record the number of glass marbles buried by each mouse and calculate the percentage of buried marbles out of the total number.

1. *Elevated zero-maze test (EZM)*

In the afternoon (from 3:00 pm to 5:00 pm) of AS 2 d, the EZM was employed to assess anxiety-like behavior in mice (Yasumoto et al., 2021). The Zero-maze used in this study consists of a circular platform with a diameter of 50 cm and a width of 10 cm. The platform is elevated 70 cm above the ground. The maze is divided into four equal quadrants, with two opposite quadrants enclosed by black walls approximately 30 cm high, forming dark enclosed spaces. The remaining two opposite quadrants are left without walls, creating bright open spaces. To experiment, a mouse is placed at the center of one of the enclosed quadrants and given 5 min to freely explore the maze. An overhead video tracking system (XR-XO206) is utilized to automatically record the time spent by the mouse in the open spaces and the number of head dips towards the edges of the open areas.

1. *Open field test (OFT)*

On the morning (from 9:00 am to 11:00 am) of the 3rd day after AS, the OFT was conducted to assess exploratory behavior and general activity in rodents (Kraeuter et al., 2019a). The OFT chamber is composed of a white plastic board measuring 40 cm × 40 cm, with white plastic walls standing at a height of 40 cm. To conduct the test, a mouse is placed at the center of the chamber and given 5 min to freely explore its surroundings. The behavior of the mouse is recorded using a video-tracking system (XR-XZ301). Various parameters are meticulously measured during the test, including the time spent in the central area defined as a 20 × 20 cm region from the chamber's center, grooming durations, total distance moved, and rearing frequency. These measures provide valuable insights into the baseline activity levels and emotional state of each mouse.

1. *Y maze test (YMT)*

On the morning (from 9:00 am to 11:00 am) of four days following AS, the YMT was performed to assess spatial working memory, which is a component of short-term memory (Kraeuter et al., 2019b). The Y-shaped maze utilized in this study was comprised of three arms arranged at 120° angles, with each arm measuring 40 cm in length, 14 cm in width, and 15 cm in height. To experiment, mice were gently placed at the center of the maze and allowed to explore freely for 8 min. An entry was counted when all four limbs of the mouse were within an arm. The total number of arm entries, indicating spontaneous locomotor activity, and successful alternations, defined as consecutive entries into all three arms without repetition, were meticulously recorded. Data collection was performed by an experimenter who was blind to the experimental conditions and tracked using a video-tracking system (XR-XY1031). Criteria for exclusion from further analysis included less than 12 total arm entries or a failure to make new entries for over 120 s during the test. However, no mice were excluded from this particular experiment. The percentage of alternation was calculated as the number of successful alternations divided by the total possible alternations (total arm entries - 2) multiplied by 100.

1. *Fear conditioning (FC) test*

On the morning (from 9:00 am to 11:00 am) of five days after AS, the mice began to undergo the FC test to evaluate their associative learning and memory related to aversive experiences (Shoji et al., 2014). During the training session of the FC test, the mice were exposed to a paired tone (30 s, 75 dB, 3 kHz) and foot shock (2 s, 0.80 mA) stimulation, followed by a 180-second adaptation period. The foot shock was initiated in the last 2 s of the tone stimulation and ended simultaneously with the tone. Following the shock presentation, the mouse remained in the chamber for an additional 30 s without any stimulation before being returned to its original home cage. After a 24-hour interval, a contextual FC test was conducted to evaluate long-term contextual fear memory, which is dependent on the hippocampus. This involved placing the mice back into the same training chamber for 5 min without the paired tone-foot shock stimulation. To assess hippocampus-independent cued fear memory, another test was conducted 24 h after the contextual FC test using a modified novel chamber. Each mouse was gently introduced into the novel chamber and allowed to freely explore for 3 min without any stimulation. This was followed by an additional 3 min where a tone (30 s, 75 dB, 3 kHz) was played. The freezing behavior exhibited by the mice during the tests was automatically monitored using a video-tracking system (XR-XC404). Freezing behavior was defined as the absence of visible movement, except for respiration, detected as immobility periods exceeding 0.75 s, reflecting the acquisition and recall of the aversive experience endured. The percentage of time spent exhibiting freezing behavior during each session was analyzed.

***In Vivo fiber photometry calcium recording***

Fiber photometry is a calcium imaging method for detecting neural activity. By using stereotaxic mouse brain atlases (2nd edition) and pre-experiments, the injection sites of the virus and placement of the fiber optic cannula were determined. The target location in the right hippocampal CA3 region was AP: - 2.54 mm, ML: - 2.85 mm, DV: - 3.00 mm. The virus, rAAV9-hSyn-GCaMP6s (2×10^12^ vg/mL, 500 nL, BrainVTA CO., Ltd., Wuhan, China), was injected into the hippocampal CA3 region with a dilution of PBS at a 1:1 volume ratio under a stereotaxic apparatus. The parameters of the injection pump were set to 500 nl volume and a speed of 50 nL/min to start virus injection. After injection, it was left for 10 min before lifting the microinjection syringe (R480, Nanoliter injection pump, RWD Life Science Co., Ltd). After 2 weeks of expression of the virus, the fiber optic cannula was implanted 200 μm above the virus injection site and then was carefully secured with dental cement. The optical fiber (200 μm O.D., NA = 0.39, 5.0 mm long) was coupled with a ferrule (10.5 mm long and 2.5 mm in diameter). A frequency-modulated 473- and 410-nm light-emitting diodes was used to stimulate calcium ion-dependent and isosbestic emission, respectively, and the signal was digitized at 6 kHz. The GCaMP6 signals were collected by using a photoreceiver system (R820, RWD Life Science CO., Ltd., Shenzhen, China). To extract the freezing behavior, a blinded evaluator manually inspected each video and recorded relevant events. F_0_ is the baseline fluorescence signal averaged over a 2-s baseline time window before the freezing behavior. The data of △F/F was generated from the recording system. △F/F values are presented as heat maps. The average and maximum of △F/F as well as the area under the curve (AUC) were analyzed during the FC test.

***Transmission electron microscope (TEM)***

After anesthetizing the mice with isoflurane, PBS and 4% paraformaldehyde (PFA) were sequentially perfused through the left ventricle. The brain was then harvested. The hippocampal tissue samples about 1 mm³ from the CA1 stratum radiatum were collected using a razor blade under a mouse brain matrix. The samples were soaked in a 2.5% glutaraldehyde (P1126, Beijing Solarbio Science & Technology Co., Ltd, Beijing, China) for electron microscopy fixation for 24 h at 4°C. Subsequently, they were post-fixed with 1% osmium tetroxide, dehydrated with a gradient of 50%, 70%, 80%, 90%, and 100% acetone, infiltrated with epoxy resin and its hardening agent, and embedded. Wuhan Servicebio Technology Co., Ltd. performed block trimming, prepared ultra-thin sections (70 nm), stained them, and captured images using transmission electron microscopy. Synaptic structural analyses were performed at 60000x magnification. Synapses were confirmed by clearly visible synaptic vesicles and postsynaptic density (PSD). Ten synapses per mouse were selected for measuring the thickness of PSD.

***Nissl Staining***

To verify the correct placement of cannulas used in our studies, brains from 20-22-month-old mice were harvested. The procedure involved anesthetizing the mice with isoflurane and performing transcardial perfusion using PBS, followed by cold 4% PFA. The brains were carefully extracted and cut coronally into 5 mm sections including the cannula location. These brain sections were then post-fixed in 4% PFA for 24 h at 4°C, rinsed with PBS, and subjected to a graded series of ethanol dehydration (30%, 50%, 70%, 80%, 90%, 95%, and 100%). Each step lasted 1 h, followed by immersion in n-butyl alcohol three times at room temperature (RT). Subsequently, the sections were immersed in paraffin at 58°C for 4 h, with regular paraffin refreshing. Afterward, the sections were embedded in fresh paraffin at 60°C, cooled on a cryo-stage, and stored at RT. Slices of the brain sections with a thickness of 8 μm were obtained using a rotary microtome (RM2125, Leica Biosystems, Baden-Wurttemberg, Germany) and dried overnight at 37°C. Deparaffinization was carried out by immersing the slices in two xylene baths for 10 min each, followed by rehydration through a descending ethanol series (100%, 95%, 90%, 80%, 70%, 50%) and distilled water (2 min for each step). Nissl staining was performed utilizing 0.1% cresyl violet (C0117, Beyotime, Shanghai, China) according to the manufacturer's protocol. After mounting the slices with neutral balsam and coverslips, and air-drying in a hood, they were imaged using a microscope (BX53M, Olympus, Tokyo, Japan) at a magnification of 4×.

***Golgi staining***

Two hours after the cued FC test, the mice's brains were harvested under isoflurane Anaesthesia, washed out of blood with milli-Q water, and processed using the FD Rapid GolgiStain^TM^ Kit (FD Neurotechnologies, Inc., Columbia, MD, USA) according to the manual and our earlier study (Jia et al., 2016). Briefly, the brain was rinsed in a fresh mixture of solution A (3.0 ml) and solution B (3.0 ml) for two weeks in the dark at RT. The mixture solution was replaced after the initial 24 h of immersion. Following this step, the tissue was transferred to solution C and stored in the dark at 4°C for one week, with a replacement of the solution after the first 24 h. Subsequently, slices with a thickness of 110 μm were sectioned using a vibratome (VT1200, Leica) and dried in the dark at RT. Next, the sections were washed twice for 3 min each with milli-Q water and then placed in a mixture (50 ml) consisting of solution D, solution E, and milli-Q water, with a volume ratio of 1:1:2. This mixture was allowed to interact with the sections for 10 min. Following this, the sections were rinsed twice for 4 min each with milli-Q water, dehydrated using graded ethanol, cleared with xylene, and finally covered with a slide using permount. Dendritic morphology in the CA1 region and pyramidal neurons were observed under OLYMPUS cellSens dimension software under microscopy (BX53M, Olympus). In the hippocampal CA1 region, both apical and basal dendrites of representative pyramidal neurons were analyzed. Neuron tracings and quantification of dendritic total length, number of branching points, and intersections at 25 μm intervals from the soma center were performed using NeuronJ (version 1.4.3) and Sholl analysis (version v3.4.4, NIH, Bethesda, MD). Data from 8 neurons per animal were averaged (n = 3 or 4). Different morphological spine types were categorized as previously described, classified as immature (including stubby, thin, and filopodia spines) and mature (including mushroom and branched spines) based on spine head and neck diameters(Havekes et al., 2016).

***F- and G-actin quantitative analysis***

Filamentous (F)- and globular (G)- actin in the hippocampus were detected by G-actin/F-actin in vivo assay kit (BK037, Cytoskeleton, CO, USA) following the manufacturer's protocol. The samples were quickly homogenized in LAS2 buffer at 37°C, which had been prepared in advance. The LAS2 buffer consisted of lysis and F-actin stabilization buffer, a stock solution of 100 mM ATP, and an EDTA-free protease inhibitor cocktail. After homogenization, the samples were incubated at 37°C for 10 min and then centrifuged at 37°C for 1 h at 100,000 × g to pellet the insoluble F-actin, leaving soluble G-actin in the supernatants. The supernatants (containing G-actin) were gently transferred to fresh tubes and F-actin depolymerization buffer was added to the pellets (containing F-actin). They were then incubated on ice for 1 h, while gently blowing with a pipette up and down every 15 min. After incubation, the samples were centrifuged at 4°C for 30 min at 15,000 g. The resulting supernatants were harvested, and aliquots were taken for protein determination using the BCA procedure. All samples were solubilized with 5× loading buffer (P0015L, Beyotime) and subjected to actin quantitation through SDS-PAGE and western blot (WB) analysis. The representative bands were analyzed by Image J and presented as the ratio of F- to G-actin.

***Co-immunoprecipitation (Co-IP)***

The fresh hippocampus was collected, washed with cold PBS, and then homogenized using a modified NP-40 lysis buffer. The lysis buffer consisted of 50 mM Tris-HCl (pH = 7.4), 1% NP-40, 0.25% Sodium deoxycholate, 150 mM NaCl, 1 mM Na_3_VO_4_, 0.1% SDS, 1 mM NaF, 1 mM PMSF, and freshly added 1× cocktail protease inhibitors. The homogenate was incubated on ice for 10 min, followed by centrifugation at 12,000 rpm for 20 min at 4°C. The resulting supernatant was collected, and the protein concentration was determined using the BCA Protein Assay kit to ensure consistency among groups. For pre-cleaning, twenty-five microliters of magnetic beads (#88804, Classic Magnetic IP/Co-IP Kit, Thermo Scientific™) were utilized. The supernatants were collected again on a magnetic stand to remove the beads. For Co-IP, a specific NgR1 antibody (10 μg) or an equivalent amount of non-specific control immunoglobulin (IgG, 10 μg) was added to the supernatant (1000 μg) and incubated overnight at 4°C on a rotator. Pre-washed 250 μL magnetic beads were then added and incubated for 1 h at room temperature on a rotator. Afterward, the beads were collected on a magnetic stand, washed three times with cold IP lysis buffer (without protease inhibitor), and resuspended in IP lysis buffer. The samples were then eluted with 100 μL of 5× lane marker sample buffer (diluted five-fold with purified water) containing DTT (50 mM) at 37°C for 20 min and gently mixed several times. Subsequently, 20 μL of each sample was loaded onto 4-12% SDS-PAGE gels for WB analysis to detect the content of binding complexes between NgR1 and NogoA.

***Isolation of hippocampal synaptosome***

The preparation of hippocampal synaptosomes was conducted following the instructions provided in the Syn-PER™ Synaptic Protein Extraction Reagent manual (87793, ThermoFisher Scientific). All steps were performed on ice to maintain sample integrity. Freshly prepared Syn-PER™ reagent was supplemented with a Mini EDTA-Free Protease Inhibitor Cocktail Tablet and 1 mM NaF as a phosphatase inhibitor. This modified mixture was added to either high-density neuron cultures or a Dounce tissue grinder containing fresh hippocampi from 2 mice. The tissues were homogenized by performing 10-12 up and down strokes, and the resulting homogenate was transferred to new 2 mL centrifuge tubes. The homogenate was then subjected to centrifugation at 1,200 g for 10 min at 4°C, and the pellet obtained was discarded. Subsequently, the supernatant was further centrifuged at 15,000 g for 20 min at 4°C. After careful removal of the supernatant, the resulting pellet was resuspended in 150 μL of lysis buffer. The lysis buffer consisted of 100 mM NaCl, 20 mM HEPES, 1 mM EDTA, 1 mM dithiothreitol, 1.0% Triton X-100, 1 mM Na_3_VO_4_, and a protease inhibitor. The resuspended homogenates were incubated with gentle rocking for 10 min at 4°C, followed by centrifugation at 10,000 g for 15 min at 4°C. The resulting supernatant, enriched with soluble proteins, was collected. Protein quantification was performed, and the concentration was adjusted to 1 or 2 μg/μL using the lysis buffer for subsequent WB analysis.

***RhoA pull-down activation analysis***

Active RhoA in the fresh hippocampus was detected by a specific RhoA Activation Assay Kit (ab211164, Abcam, Cambridge, UK) following the instructions provided in the manual. Briefly, the fresh hippocampus was washed with ice-cold PBS and then homogenized on ice using a Polytron homogenizer in 800 μl of 1× assay buffer. The homogenate was placed on ice for 5 min and subsequently centrifuged at 4°C for 10 min at 14,000 g to remove insoluble materials. The resulting supernatant was collected in a new tube, and the protein concentration was determined using a bicinchoninic acid (BCA) protein assay kit (E162-05, GenStar, Beijing, China). The sample was adjusted to a concentration of 1 mg/mL with 1× assay buffer. Rhotekin RBD agarose beads (to pull down the active form of RhoA) were added to each tube at a ratio of 1:25. The tubes were then incubated at 4°C for 1 h with gentle agitation, followed by brief centrifugation for 10 s at 14,000 g. The beads pellets were collected, washed three times with 500 μL of 1× assay buffer, and all supernatant was removed. The pellets were then resuspended in 2× reducing SDS-PAGE sample buffer, boiled for 5 min at 95°C, centrifuged for 10 s at 14,000 g, cooled on ice, and stored at -20°C for further WB analysis.

***qRT-PCR***

In this study, various brain regions were quickly harvested from mice, including the hippocampus, prefrontal cortex (PFC), amygdala, neocortex, striatum, and cerebellum. These samples were obtained from both the Control (Con) group and the groups at 1, 3, and 7 d after AS to detect transcript expression of NgR1, NogoA, MAG, and OMgP.

For the process of hippocampal subregions’ segmentation, the hippocampi from wild-type aged mice were rapidly harvested. The hippocampi were individually dissected by first cutting perpendicularly to the longitudinal axis along the dorsomedial hippocampus, and then along the hippocampal fissure, all under a stereomicroscope (SZX16, Olympus). Following this, the CA3 region was isolated by cutting along the edge of the DG, while the CA1 and DG were separated by cutting along the hippocampal fissure as detailed in a previous study(Fanselow & Dong, 2010). Based on previous research, the CA1 region showed enrichment of Nephroblastoma Overexpressed (Nov) and Wolfram Syndrome 1 (Wfs1) genes, while the CA3 region exhibited prevalence of Iodotyrosine Deiodinase (Iyd) and Sparcosteonectin, CWCV, and Kazal-like Domains Proteoglycan (Spock1). In the DG area, Desmoplakin (Dsp) and T-cell Lymphoma Invasion and Metastasis 1 (Tiam 1) were found to be predominant. To extract total RNA, we utilized the RNeasy Mini Kit (74104, Qiagen), followed by DNaseI treatment. Assessment of RNA quality and quantity was conducted using a Nanodrop Spectrophotometer (ThermoFisher Scientific) with an acceptable absorbance ratio at 260/280 nm ranging between 1.8-2.2. For mRNA expression analysis, 1.0 μg of total RNA was used to generate cDNA through reverse transcription employing the SuperscriptTM IV Reverse Transcriptase enzyme (18090010, ThermoFisher Scientific). Quantitative real-time PCR was performed using a LightCycler 1.5 (Roche Diagnostics GmbH, Mannheim, Germany) in conjunction with the QuantiTech SYBR Green PCR kit (Qiagen, Hilden, Germany), following the manufacturer's protocol. A primer pair concentration of 10 μM was utilized. The data were represented as 2−ΔΔCT and normalized to the expression of *gapdh*.

The forward (F) and reverse (R) primer sequences (5'→3') used in this study are as follows. *ngr1* (F: GCCTGGAGGGTAGCAACAC; R: CGTGAGAGATTCGGTTGCCA), *nogoa* (F: TCGGGCTCAGTGGATGAGA; R: ACAGTGTTACCTGGCTGCTC), *mag* (F: AGCAGAAGGTGCAGAAGTCA; R: AGTGGCCTTTCAACCAAGTCT), *omgp* (F: CGACTCCCACAACAAGACGA; R: AAGATGCCAGGCGTGAGAAA), *Nov* (F: AGTGCCCCAGTATCACCGA; R: GTCACAGGGTCTCATCTCAGA), *Wfs1* (F: CGGGAAGAAACGGACAGAGC; R: CGTAGGTAGTGTTTGCCCAC), *Iyd* (F: ACCCGATACCCAGAACAGGA; R: AGCTGTTCCTGCTGCTTTGA), *Spock1* (F: CGTACACGTCCAAGTGCAAGT; R: CTTGATGACTCTATTGGCGTCC), *Dsp* (R: GCATTCTTCTAGGGAGACTCAGT; F: TCCACTCGTATTCCGTCTGGG), *Tiam1* (F: GAAGATGGAGTAAGACTGGTCCC, R: CAGAAGGCACTGTAGAGCTTG), and *gapdh* (F: ATGTGTCCGTCGTGGATCTG; R: AGTTGGGATAGGGCCTCTCTT).

**Ex vivo experiments**

***Acute hippocampal slice for electrophysiology***

Transverse hippocampal slices with a thickness of 300 μm were prepared from adult male C57BL/6 mice that were 8 weeks old. The preparation method described in previous studies was followed, with some moderate modifications (Li et al., 2022). Each mouse was administered either LPS (5 μg / 2 μL, L4391, sigma) or saline (2 μL) via intracerebroventricular (i.c.v.) injection. After a 12-hour post-injection period, transverse hippocampal slices were dissected in an ice-cold dissection buffer that was bubbled with a mixture of 95% O_2_ and 5% CO_2_. The dissection buffer composition consisted of 212.7 mM sucrose, 2.6 mM KCl, 26 mM NaHCO_3_, 1.23 mM NaH_2_PO_4_, 10 mM dextrose, 1 mM CaCl_2_, and 3 mM MgCl_2_. Subsequently, the slices were allowed to recover and incubated at RT for 60 min in artificial cerebrospinal fluid (ACSF), which contained 124 mM NaCl, 5 mM KCl, 26 mM NaHCO_3_, 1.25 mM NaH_2_PO_4_, 10 mM dextrose, 2.5 mM CaCl_2_, and 1.5 mM MgCl_2_, which was continuously bubbled with a mixture of 95% O_2_ and 5% CO_2_. Recordings were conducted in a submersion chamber that was perfused with ACSF at a temperature of 30 ± 1 °C, with a flow rate of 2 mL/min. To record field excitatory postsynaptic potentials (fEPSPs), a borosilicate glass micropipette (2-3 MΩ) filled with ACSF was positioned at a depth of 150-200 μm in the stratum radiatum of the hippocampal CA1 region. A concentric bipolar stimulating electrode in the stratum radiatum of the CA1 region was used to stimulate the Schaffer collaterals. Baseline stimulation was set at 40% of the maximum fEPSP slope, and pulses (0.2 ms, 0.03 Hz) were applied for baseline recordings to monitor the fEPSP slope over time. After achieving a stable baseline for 20 min, long-term potentiation (LTP) was induced using three high-frequency stimulation (HFS) trains with parameters of 100 Hz, 1 s duration, and a 10-second interval between trains, all at the same intensity. The magnitude of LTP was calculated as the average response recorded 50-60 min after HFS, normalized to a baseline fEPSP slope size set at 100%. Paired-pulse facilitation (PPF) was measured using a 50 ms interstimulus interval. For the intervention experiments, Nogo-P4 (4 μM, Alpha Diagnostic Intl Inc., San Antonio, TX, USA), NEP1-40 (300 mM,1984, R&D Systems), and jasplakinolide (200 nM, 2792, R&D Systems) were applicated to hippocampal slices via the perfusion line in ACSF. Statistical analysis was performed using GraphPad Prism 9.0 (version 9.4.0) with a one-way ANOVA followed by Tukey’s post hoc test for multiple comparisons.

**In vitro experiments**

***Primary culture of hippocampal neurons***

Primary cultures of hippocampal neurons were dissociated from P0 SD neonatal rats. Previous studies have shown no significant differences in synaptic properties between mouse and rat hippocampal neurons(Li et al., 2022). The hippocampi were isolated from fetuses in cold DMEM (SH30284-01, Fisher Scientific, Ottawa, Ontario, CAN) and dissociated using diluted 0.125% trypsin-EDTA (25200056, ThermoFisher Scientific) at 37°C for 15 min, The digestion was terminated, and the cells were resuspended in a mixed neuronal medium composed of neurobasal medium (A35829-01, Invitrogen, ThermoFisher Scientific), supplemented with 2% B27 (17504-044, Gibco^TM^, ThermoFisher Scientific), 5% fetal bovine serum (FBS, 10100-147, Gibco^TM^, ThermoFisher Scientific), penicillin/streptomycin (100 U/mL), and 2% GlutaMAX^TM^ (35050061, Gibco^TM^, ThermoFisher Scientific). The cell suspensions were passed through a Falcon 70 μM cell strainer (352350, Corning, NY, USA), centrifuged at 1000 g for 3 min, and resuspended again in the mixed neuronal medium. The cell count was determined using a sterile trypan blue (T8154, Merck Millipore, Darmstadt, Germany) on a glass cell counting chamber under a light microscope. The cells were then diluted in the mixed neuronal medium. For biochemical experiments, the diluted cells were plated at a density of 2.5 × 10^5^ cells/cm^2^ on plastic plates of 12-well dishes. For immunocytochemical experiments, the diluted cells were plated at a density of 5.0 × 10^4^ cells/cm^2^ on 12-well plates with 18-mm-diameter glass coverslips pre-coated with poly-L-lysine (PLL, 100 μg/mL, P4832, Sigma). After incubating at 37°C in a 5% CO_2_ atmosphere for approximately 4 h to allow for neuronal attachment, the mixed neuronal medium was replaced with a neuronal culture medium. The neuronal culture medium consisted of neurobasal medium supplemented with 2% B27, 1% penicillin/streptomycin (10378016, ThermoFisher Scientific), 1% GlutaMAX^TM^, 1% NaCl (3.75M), and 1% D-glucose (30%) supplement. The neurons were cultured at 37°C in a 5% CO_2_ humidified atmosphere, with half of the medium in each well replaced every 3 d. Experiments were conducted on the neurons at 15-16 days in vitro (DIV15-16). On the third day, AraC (2.5 mM, 1000x, C1768, Sigma) was added to the cultures by replacing half of the medium with fresh medium.

***Neuron/microglia transwell co-culture system***

The neuron/microglia transwell co-culture system was established as follows (Chang et al., 2024). BV2 microglial cells purchased from KALANG Biological Technology Co., Ltd (KL-0493, Shanghai, China) were cultured in DMEM (12430054, Gibco^TM^, ThermoFisher Scientific) supplemented with 10% FBS, 100 IU/mL penicillin, and 100 mg/mL streptomycin. For the transwell experiments, 2 × 10^4^ BV2 cells per well were seeded into transwell insert chambers (3460, 0.4 μm membrane pore size, Corning) pre-coated with PLL. These chambers were compatible with 12-well plates, creating an insert co-culture system that mimicked in vivo conditions. At DIV14-15 of hippocampal neuronal cultures, BV2 cells were pre-treated and prepared with lipopolysaccharide (LPS, 1 μg/mL dissolved in PBS, L4391, Sigma) for 12 h. Afterward, the medium was changed to remove any remaining LPS residues before co-culturing with neurons. Upon reaching DIV15-16, the transwell inserts containing either activated or inactivated BV2 cells were placed into the corresponding neuronal culture plates for a 24-hour co-culture, each maintained in their respective culture media. For specific treatments, Nogo-66/Neurite outgrowth inhibitory peptide Nogo-P4 (4 μM) and NEP1-40 (1 μM) were incubated in neuronal cultures for 24 h. Additionally, the F-actin stabilizer jasplakinolide (1 μM) was applied for 60 min. Following the treatment period, the medium was discarded, and the cells were washed three times with warm PBS before proceeding to WB analysis or fixation for Immunocytochemistry (ICC).

**In vivo and in vitro sample analysis**

***WB analysis***

In this study, tissues or cultured neurons were homogenized using RIPA lysis buffer (50 mM Tris-HCl, 1% Triton X-100, 150 mM NaCl, 0.1% SDS, 0.5% Sodium deoxycholate, 10 mM NaF, 1 mM PMSF), supplemented with a protease inhibitors cocktail tablet. The homogenization process was carried out under gentle rocking for 10-20 min at 4°C. Following homogenization, the lysates were post-centrifuged at 10,000 g for 20 min at 4°C. Protein concentrations in the supernatants were determined using the BCA protein assay kit. Protein samples were adjusted to concentrations of 1 or 2 μg/μL in protein lysis buffer, supplemented with 5× sample loading buffer and 50 mM DTT. The samples were then boiled at 95°C for 5 min and stored at -20°C to ensure uniformity for subsequent immunoblot analysis. For protein separation, equal amounts of proteins from each group were loaded onto 4-12% or 4-20% Omni-PAGE™ Hepes-Tris gels (Epizyme Biotech, Shanghai, China) and transferred to 0.22 μm PSQ or 0.45 μm PVDF membranes based on molecular weight. The membranes were blocked with 5% non-fat milk or 5% BSA (for phosphorylated proteins) in Tris-buffered saline with 0.1% Tween-20 (TBST) for 1 h at RT. Subsequently, the membranes were incubated with primary antibodies (as listed in Table 1) diluted in primary antibody dilution buffer (P0023A, Beyotime) or BSA. The primary antibody incubation was carried out overnight at 4°C, followed by three 5-minute washes with TBST. Then, the membranes were incubated with species-appropriate secondary antibodies diluted in 5% non-fat milk or 5% BSA in TBST for 1 h at RT. The secondary antibodies used included goat anti-rabbit IgG (1:10,000, A0208, Beyotime), goat anti-mouse IgG (1:10,000, A0216, Beyotime), and rabbit anti-goat IgG (1:10,000, BS30503, Bioworld Technology co, Ltd., Nanjing, China). After incubation, the membranes were washed three times for 5 min each with TBST and developed using enhanced chemiluminescence reagents (P0018A, Beyotime). Densitometric analysis of the membranes was performed using Fiji software (NIH, Version 2.3.0/1.53q, Bethesda, MD, USA).

***Immunofluorescence (IF) and Immunocytochemistry (ICC) assay***

Mice were anesthetized using isoflurane and transcardially perfused with PBS, followed by 4% paraformaldehyde (PFA) for immunofluorescence (IF). Some mice underwent the contextual FC test lasting approximately 90 min to facilitate fosB and RhoA-GTPase (as listed in Table 1) staining. The brains were then immersed in 4% PFA for further post-fixation, dehydrated in 30% sucrose, and embedded in an OCT compound (Tissue-TEK, 4583, Sakura, Torrance, CA, USA). The brain tissue was stored at -80°C until further processing. Coronal sections of the hippocampus were cut at 30 μm thickness using a frozen section machine (CM1950, Leica). The brain slices were washed with PBS and, when necessary, permeabilized with 0.3% Triton X-100. Subsequently, they were blocked in 5% or 10% normal donkey serum (abs935, absain, Shanghai, China) in PBS with or without 0.3% Triton-X100 and incubated overnight at 4°C with primary antibodies (as listed in Table 1). For PSD95 staining, 8 μm paraffin slices were utilized and processed similarly to Nissl staining, including antigen retrieval in a sodium citrate solution. These slices were then incubated with the PSD95 antibody at 4°C for 48 h. After washing, the slices were incubated with appropriate secondary antibodies, such as goat anti-rabbit IgG Alexa Fluor 488 (1:500, ab15007, Abcam), donkey anti-rabbit IgG Alexa Fluor 488 (1:500, ab150073, Abcam), donkey anti-goat IgG Alexa Fluor 647 (1:500, ab150131, Abcam), and donkey anti-mouse IgG Alexa Fluor 555 (1:500, ab150106, Abcam) at the specified dilutions, respectively. Following another round of washing, the slices were mounted with superKineTM enhanced antifade mounting medium with DAPI (BMU107-CN, Abbkine Scientific Co., Ltd, Wuhan, China) and imaged using Olympus (FV1000) and Zeiss (LSM 880) confocal microscopes at 40× magnification.

To stain neurons for NgR1, PSD95, s-GluA1, s-GluA2, and βIII-tubulin, the following protocol was followed. Neurons were washed, fixed, and permeabilized as required. Blocking was performed using 10% donkey serum in PBS with or without 0.3% Triton-X100. Subsequently, the neurons were incubated with primary antibodies (as specified in Table 1) and appropriate secondary antibodies. The secondary antibodies used were donkey anti-goat IgG Alexa Fluor 488 (1:500, ab150129, Abcam), donkey anti-mouse IgG Alexa Fluor 488 (1:500, ab150105, Abcam), goat anti-chicken IgG Alexa Fluor 647 (1:500, ab150171, Abcam), and goat anti-guinea pig IgG Alexa Fluor 555 (1:500, ab150186, Abcam). F-actin staining was achieved using phalloidin-iFluor-647 without employing a secondary antibody. Finally, coverslips were mounted with superKineTM enhanced antifade mounting medium with DAPI (Abbkine, Atlanta, GA, USA). The stained samples were imaged using a Zeiss confocal microscope at either 63× or 63×/zoom 1.6 magnification, with a Z-stack of 0.75 μm and a resolution of 1024 × 1024 pixels, respectively.

***Measurements for hippocampal membrane protein***

To analyze membrane protein expression in neuronal cultures, a surface biotinylation pull-down method was employed. Sulfo-NHS-SS-Biotin (PG82077, ThermoFisher Scientific) was prepared fresh in PBS with 1 mM CaCl_2_ and 0.5 mM MgCl_2_ at a concentration of 1 mg/mL. This solution was added to the high-density neurons and incubated for 30 min at 4°C with gentle shaking to label all surface membrane proteins. The biotinylated cells were then homogenized in RIPA buffer and centrifuged at 13,200 rpm for 20 min at 4°C. The resulting supernatant was collected and transferred to a new tube. Protein concentration was determined using a BCA Protein Assay kit, and adjustments were made to ensure uniformity across different groups. Next, 50-100 μL of neutravidin agarose beads (29202, ThermoFisher Scientific) in RIPA buffer were added to 500 μg of protein lysate and incubated for 2 h at 4°C with rotation. The beads were subsequently washed three times with cold RIPA buffer. Bound proteins were eluted in 5× SDS reducing buffer and left at RT for 30 min. The supernatant containing the eluted proteins was transferred to a new tube for subsequent WB analysis.

For hippocampal tissues, the extraction of hippocampal membrane fractionation was performed according to the manual of the Plasma Membrane Protein Extraction Kit (ab65400, Abcam). Briefly, the hippocampus from 3 mice was used as a sample and homogenized in a Dounce tissue grinder including 2 volumes of the homogenized buffer with protease inhibitor cocktail and fresh PMSF (the final concentration was 1 mM, until it was completely lysed. Then the homogenate was transferred to 2 ml tubes and centrifuged by 700 × g for 10 min at 4°C. The supernatant was collected and recentrifuged by 14,000 × g for 30 min at 4°C. The pellet was collected and re-suspended with the upper phase solution and then mixed with the lower phased solution for a 5-minute incubation on ice. After being recentrifuged by 1,000 × g for 5 min at 4°C, the upper phase solution was carefully collected in a new tube. To precipitate the membrane proteins as much as possible, the above procedure was repeated for once time. The upper phase was mixed, diluted in 5 volumes of water, and incubated on ice for 5 min. After that, the tube was spined at top speed at a microcentrifuge tube for 10 minutes at 4°C. The supernatant was removed and the pellet was collected as the plasma membrane protein. The plasma membrane proteins were dissolved in 0.5% Triton X-100 in PBS with protease inhibitor cocktail and fresh PMSF, quantified by BCA Protein Assay kit, and adjusted with 5X sample buffer to a concentration of 1 μg/μL for WB assays.

**References**

Bobo-Jimenez, V., Delgado-Esteban, M., Angibaud, J., Sanchez-Moran, I., de la Fuente, A., Yajeya, J., Nagerl, U. V., Castillo, J., Bolanos, J. P., & Almeida, A. (2017). APC/C(Cdh1)-Rock2 pathway controls dendritic integrity and memory. *Proc Natl Acad Sci U S A, 114*(17), 4513-4518. doi:10.1073/pnas.1616024114

Chang, H., Li, Z., Zhang, W., Lin, C., Shen, Y., Zhang, G., Mao, L., Ma, C., Liu, N., & Lu, H. (2024). Transfer of cGAMP from neuron to microglia activates microglial type I interferon responses after subarachnoid hemorrhage. *Cell Commun Signal, 22*(1), 3. doi:10.1186/s12964-023-01362-3

de Brouwer, G., Fick, A., Harvey, B. H., & Wolmarans, W. (2019). A critical inquiry into marble-burying as a preclinical screening paradigm of relevance for anxiety and obsessive-compulsive disorder: Mapping the way forward. *Cogn Affect Behav Neurosci, 19*(1), 1-39. doi:10.3758/s13415-018-00653-4

Fang, Y., Yao, L., Li, C., Wang, J., Wang, J., Chen, S., Zhou, X. F., & Liao, H. (2016). The blockage of the Nogo/NgR signal pathway in microglia alleviates the formation of Abeta plaques and tau phosphorylation in APP/PS1 transgenic mice. *J Neuroinflammation, 13*(1), 56. doi:10.1186/s12974-016-0522-x

Fanselow, M. S., & Dong, H. W. (2010). Are the dorsal and ventral hippocampus functionally distinct structures? *Neuron, 65*(1), 7-19. doi:10.1016/j.neuron.2009.11.031

Havekes, R., Park, A. J., Tudor, J. C., Luczak, V. G., Hansen, R. T., Ferri, S. L., Bruinenberg, V. M., Poplawski, S. G., Day, J. P., Aton, S. J., Radwanska, K., Meerlo, P., Houslay, M. D., Baillie, G. S., & Abel, T. (2016). Sleep deprivation causes memory deficits by negatively impacting neuronal connectivity in hippocampal area CA1. *Elife, 5*. doi:10.7554/eLife.13424

Jia, M., Liu, W. X., Yang, J. J., Xu, N., Xie, Z. M., Ju, L. S., Ji, M. H., Martynyuk, A. E., & Yang, J. J. (2016). Role of histone acetylation in long-term neurobehavioral effects of neonatal Exposure to sevoflurane in rats. *Neurobiol Dis, 91*, 209-220. doi:10.1016/j.nbd.2016.03.017

Kraeuter, A. K., Guest, P. C., & Sarnyai, Z. (2019). The Open Field Test for Measuring Locomotor Activity and Anxiety-Like Behavior. *Methods Mol Biol, 1916*, 99-103. doi:10.1007/978-1-4939-8994-2_9

Kraeuter, A. K., Guest, P. C., & Sarnyai, Z. (2019). The Y-Maze for Assessment of Spatial Working and Reference Memory in Mice. *Methods Mol Biol, 1916*, 105-111. doi:10.1007/978-1-4939-8994-2_10

Li, Q. Q., Chen, J., Hu, P., Jia, M., Sun, J. H., Feng, H. Y., Qiao, F. C., Zang, Y. Y., Shi, Y. Y., Chen, G., Sheng, N., Xu, Y., Yang, J. J., Xu, Z., & Shi, Y. S. (2022). Enhancing GluN2A-type NMDA receptors impairs long-term synaptic plasticity and learning and memory. *Mol Psychiatry, 27*(8), 3468-3478. doi:10.1038/s41380-022-01579-7

Rolando, C., Parolisi, R., Boda, E., Schwab, M. E., Rossi, F., & Buffo, A. (2012). Distinct roles of Nogo-a and Nogo receptor 1 in the homeostatic regulation of adult neural stem cell function and neuroblast migration. *J Neurosci, 32*(49), 17788-17799. doi:10.1523/JNEUROSCI.3142-12.2012

Shoji, H., Takao, K., Hattori, S., & Miyakawa, T. (2014). Contextual and cued fear conditioning test using a video analyzing system in mice. *J Vis Exp*(85). doi:10.3791/50871

Wei, P., Jia, M., Kong, X., Lyu, W., Feng, H., Sun, X., Li, J., & Yang, J. J. (2023). Human umbilical cord-derived mesenchymal stem cells ameliorate perioperative neurocognitive disorder by inhibiting inflammatory responses and activating BDNF/TrkB/CREB signaling pathway in aged mice. *Stem Cell Res Ther, 14*(1), 263. doi:10.1186/s13287-023-03499-x

Yasumoto, Y., Stoiljkovic, M., Kim, J. D., Sestan-Pesa, M., Gao, X. B., Diano, S., & Horvath, T. L. (2021). Ucp2-dependent microglia-neuronal coupling controls ventral hippocampal circuit function and anxiety-like behavior. *Mol Psychiatry, 26*(7), 2740-2752. doi:10.1038/s41380-021-01105-1

**Table 1.** **Primary antibodies used in this study**

| **Antibody** | **Catalogue Number** | **Company** | **Host** | **Applications** |
| --- | --- | --- | --- | --- |
| Nogo-66 recptor 1 (NgR1) | AF1440  ab250758 | R&D systems  Abcam | Goat  Rabbit | WB 1:1000; IF 1:100;  ICC 1:200  CO-IP 1:100 |
| neurite outgrowth inhibitor A (NogoA) | PA5-20366  13401  sc-25660 | Thermo Fisher Scientific  (Invitrogen)  Cell signaling Technology  Santa Cruz Biotechnology | Rabbit  Rabblt  Rabbit | WB 1.5 μg/ml;  IF 10 μg/ml  WB 1:1000  WB: 1:1000;  IF 1:100 |
| Leucine-rich repeat and immunoglobulin-like domain-containing nogo receptor-interacting protein 1 (LINGO-1) | ab23631 | Abcam | Rabbit | WB 1:1000 |
| Tumor necrosis factor receptor superfamily member 19 (TROY) | sc-398526 | Santa Cruz Biotechnology | mouse | WB 1:1000 |
| Low-affinity nerve growth factor receptor (P75^NTR^) | 55014-1-AP | Proteintech | Rabbit | WB 1:1000 |
| Ras homolog family member A (RhoA) | ab187027 | Abcam | Rabbit | WB 1:1000 |
| Rho associated coiled coil containing protein kinase 1 (ROCK1) | 21850-1-AP | Abcam | Rabbit | WB 1:10000 |
| Rho associated coiled coil containing protein kinase 2 (ROCK2) | ab125025 | Abcam | Rabbit | WB 1:1000 |
| LIMK1 (phospho T508) | ab194798 | Abcam | Rabbit | WB 1:500 |
| LIM domain kinase 1 (LIMK1) | 19699-1-AP | Proteintech | Rabbit | WB 1:500 |
| cofilin 2(phospho Ser3) | ab14134 | Abcam | Rabbit | WB: 3.0 μg/ml |
| cofilin 2 | 11848-1-AP | Proteintech | Rabbit | WB 1:1000 |
| PSD95 | MABN68 | Merck Millipore | Mouse | WB 1:1000; IF 1:50;  ICC 1:100 |
| Active RhoA (RhoA-GTPase) | 26904 | Neweast Bioscience | Mouse | IF 1:500 |
| Synapsin 1 | AB1543 | Merck Millipore | Rabbit | WB 1:500 |
| glutamate receptor 1 (GluA1) | ab31232  AGC-004-GP | Abcam  Alomone labs | Rabbit  Guinea pig | WB 1:1000  ICC 1:200 |
| glutamate receptor 2 (GluA2) | Ab133477  AGC-005-GP  1194-1-AP | Abcam  Alomone labs  Proteintech | Rabbit  Rabbit | WB 1:1000  ICC 1:200  WB 1:1000 |
| glutamate receptor 3 (GluA3) | ab232887 | Abcam | Rabbit | WB 1:1000 |
| FosB | 2251S | Cell signaling Technology | Rabbit | IF 1:500 |
| GAPDH | 60004-1-lg | Proteintech | Mouse | WB 1:1000 |
| neuronal class III β-tubulin | ab78078  ab41489 | Abcam  Abcam | Mouse  Chicken | WB: 1:1000  ICC: 1:500 |
| Na+/K+ ATPase 1 (ATP1A1) | 14418-1-AP | Proteintech | Rabbit | WB 1:800 |
| β-actin | 20536-1-AP  A1978 | Proteintech  Sigma | Rabbit  Mouse | WB 1:1000  WB 1.0 μg/ml |
| α-actin | AAN01 | Cytoskeleton | Rabbit | WB 1:1000 |
| phalloidin-Alexa Fluor™ 647 | A22287 | ThermoFisher Scientific | Alexa Fluor™ 647 | ICC 1:400 |


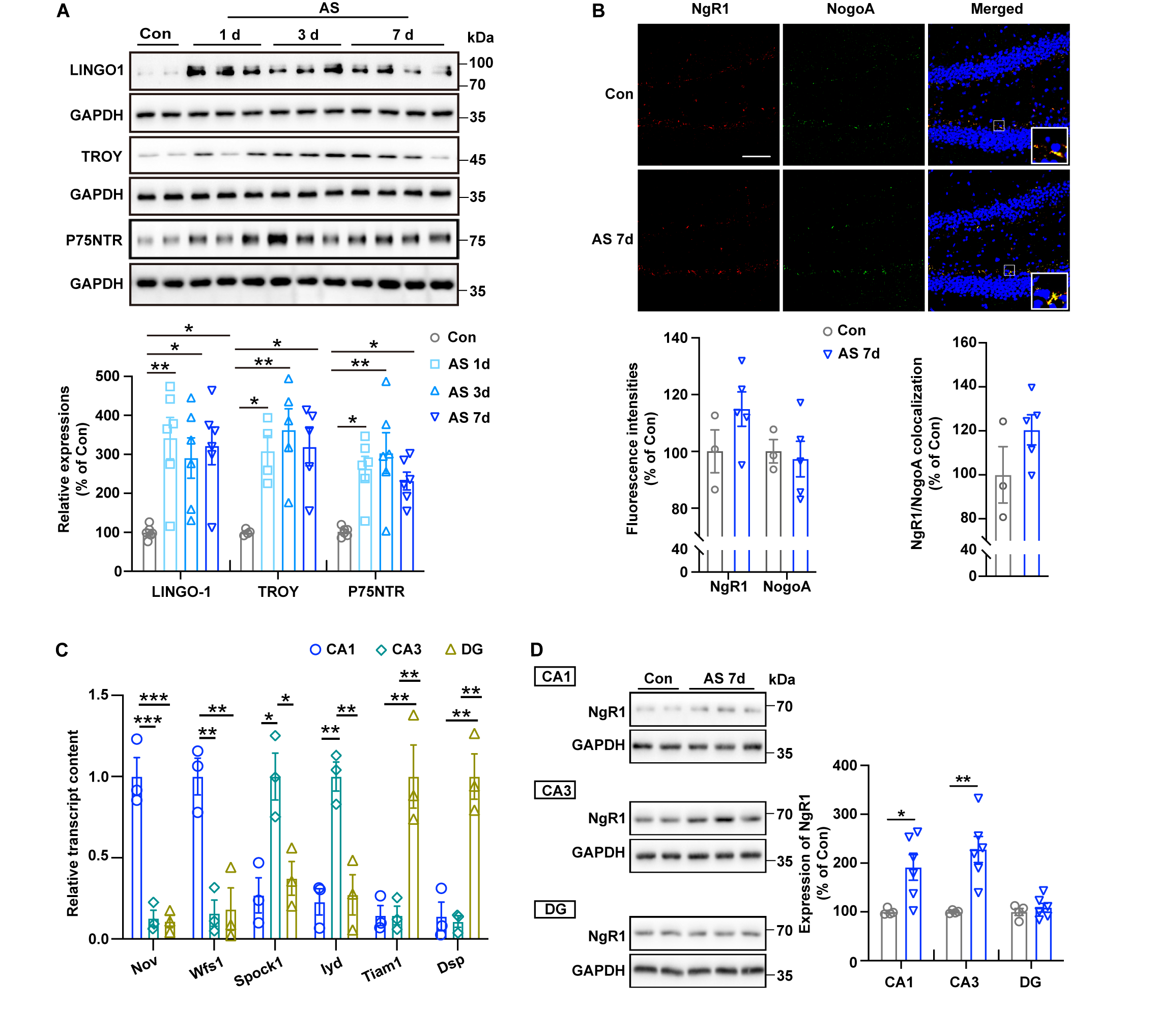


**Supplement 1. Upregulation of Hippocampal NgR1 Co-receptors in Aged Mice: Effects of Anaesthesia and surgery (AS) Treatment at 1, 3, and 7 Days. Differential Expression in CA1 and CA3 Subregions, but Not DG, at AS 7 Days compared to the Control Group**

**(A)** Expression levels of NgR1 co-receptors (LINGO1, TROY, and P75NTR) in the hippocampus at different time points in aged mice with or without AS treatment (LINGO-1 and P75NTR: n = 6;/group TROY: n = 4 of the Con and AS 1 d groups, n = 5 of the AS 3 d and 7 d groups). Comparisons were made with one-way ANOVA followed by Turkey’s post hoc test.

**(B)** Immunofluorescence (IF) images of NgR1 and NogoA in the hippocampal DG subregion of aged mice from the Con and AS 7 d groups were captured at a magnification of 40×. The relative fluorescence intensities of NgR1, NogoA, and the level of their colocalization were analyzed (n = 3 of the Con group, n = 5 of the AS 7 d group). Scale bar = 50 μm.

**(C)** Transcript enrichment analysis was conducted to assess the levels of specific transcripts in the CA1, CA3, and DG subregions of aged mice. One-way ANOVA followed by Bonferroni’s post hoc test was used (n = 3/group).

**(D)** Expression of NgR1 was detected in the CA1, CA3, and DG regions of the AS 7 d group compared to the Con group (n = 4 of the Con group, n = 6 of the AS 7 d group).

**(B, D)** The two groups were compared using an unpaired t-test with the Mann–Whitney U test. All data were presented as mean ± SEM. **P* < 0.05 and ***P* < 0.01 compared with the Con group.


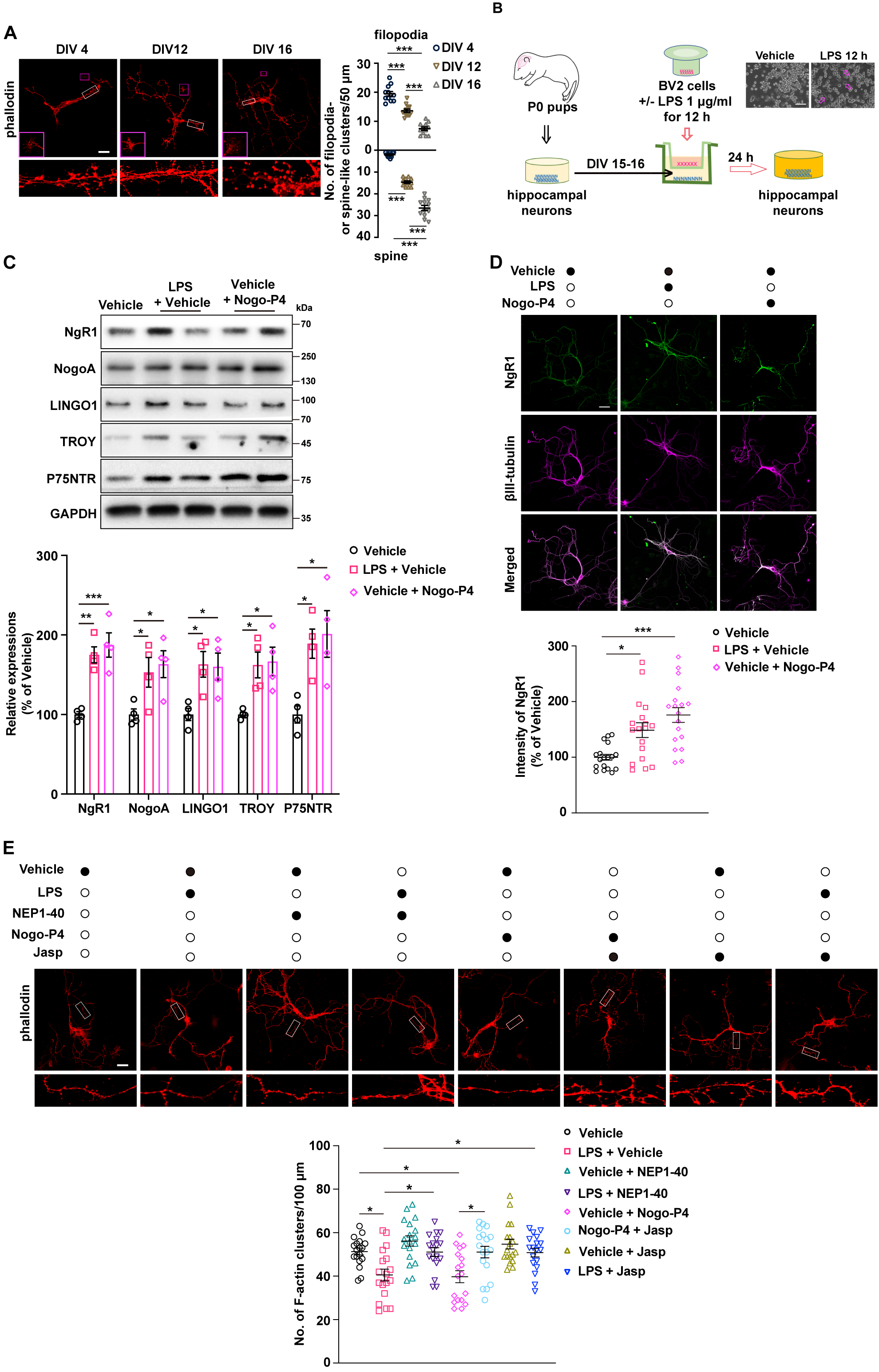


**Supplement 2. Induced overexpression of Nogo66-NgR1 signaling by co-culturing LPS pre-treated BV2 microglia led to F-actin depolymerization in hippocampal neuronal cultures: effects on Nogo-P4, NEP1-40, and Jasplakinolide intervention**

**(A)** Phalloidin staining is utilized to label F-actin in low-density cultured rat hippocampal neurons. During the developmental stages, significant morphological changes occur in the dendritic protrusions of neurons at DIV4, 12, and 16. At DIV4, when observed under higher magnification, only a few filopodia-like protrusions are observed along the dendrites. By DIV12, the protrusions exhibit diverse shapes, including filopodia-like extensions and relatively more spine-like protrusions. At DIV16, the majority of protrusions display mushroom-like or thin/stubby spine shapes, showcasing higher levels of F-actin enrichment compared to filopodia-like protrusions. Furthermore, during neuronal development, outgrowth cones can be observed. N = 8-12 neurons (2-4 neurons/dish) for different time points. The scale bar represents 20 μm.

**(B)** Experimental Design In Vitro: hippocampal neuronal cultures derived from P0 neonatal SD rats were maintained until DIV15-16. These cultures were then exposed to activated or inactivated BV2 microglia for 24 h using co-culture insert systems. The presence or absence of NEP1-40 and jasplakinolide (Jasp) treatments was initiated by adding them to the neuronal culture dishes at the start of co-cultures. For the Nogo-P4 experiment, Nogo-P4 peptide was added to the neuronal cultures at the beginning of the inactivated BV2 microglia co-culture systems. Before this, BV2 microglia cultures were pretreated with either Vehicle or LPS (for pre-activation) intervention for 12 h. Accompanying BV2 microglia images illustrate the morphological changes observed in inactivated and activated microglia. Following a 12-hour LPS pretreatment, activated microglia (indicated by magenta arrows) displayed an enlarged cell body, loss of significant ramification, and transformed into an amoeboid shape. Scale bar = 80 μm.

**(C)** The expressions of NgR1, NogoA, LINGO-1, TROY, and P75NTR were assessed via WB analysis in the hippocampal neuronal cultures following the application of Vehicle, LPS, and Nogo-P4 treatments (n= 3 of the Vehicle group, n = 4 of the LPS + Vehicle and Vehicle + Nogo-P4).

**(D)** The relative intensity of NgR1 in hippocampal neuronal cultures at DIV15 was measured following 24 h of co-cultures with the application of Vehicle, LPS, and Nogo-P4 (n= 18 neurons/group), Scale bar = 20 μm.

**(E)** The number of F-actin (labeled by phalloidin) clusters per 100 μm in hippocampal neuronal cultures at DIV16 was analyzed using ICC staining following 24 hours of co-cultures with LPS pretreated BV2 microglia cells, Nogo-P4, NEP1-40, and Jasp interventions (n= 18 neurons/group). Scale bar = 20 μm.

All data were analyzed by one-way ANOVA followed by Turkey’s post hoc test and presented as mean ± SEM. **P* < 0.05, ***P* < 0.01, and ****P* < 0.001.

**
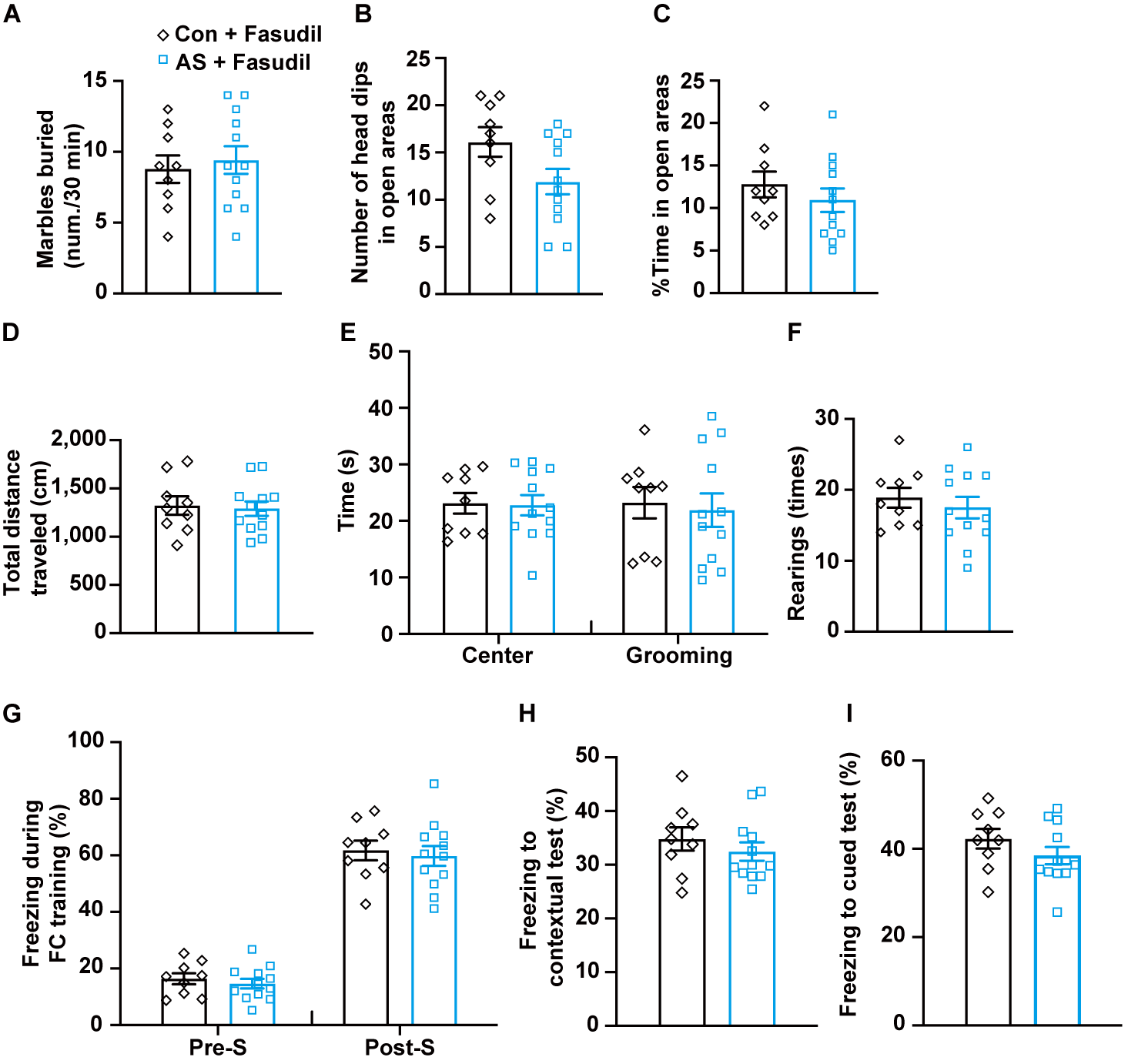
**

**Supplement 3. Behavioral assessments in aged mice exposed to Anaesthesia and surgery (AS) with a Rho kinase inhibitor Fasudil treatment**

**(A)** The number of buried marbles in the MBT was recorded for 30 min and compared between the two groups.

**(B, C)** The number of head dips and the percentage time in open areas were analyzed in the EZM.

**(D-F)** Total distance traveled, time spent in the center and grooming, and times of rearing were assessed in the OFT.

**(G-I)** The percentage of freezing behavior was analyzed during FC training, as well as during the contextual and cued FC tests.

Data were analyzed using unpaired and nonparametric t-tests, followed by the Mann-Whitney test, and are presented as mean ± SEM (n = 9 of the Con + Fasudil group; n = 12 of the AS + Fasudil group).

**
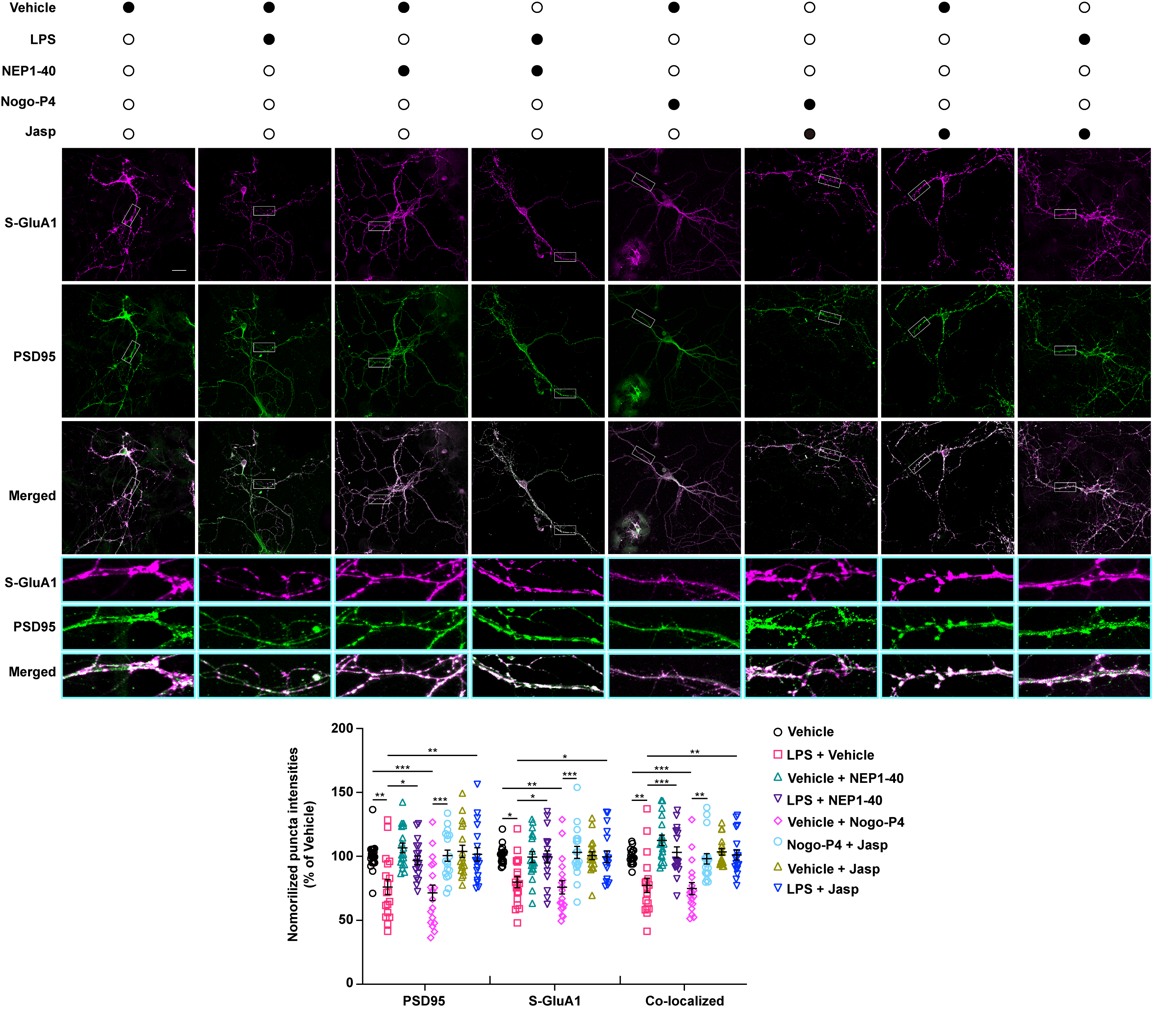
**

**Supplement 4. Effects of LPS, Nogo-P4, NEP1-40, and Jasplakinolide on the expressions of PSD95 and surface GluA1 on BV2-hippocampal neuronal co-cultures**

All data were analyzed using one-way ANOVA followed by Tukey’s post hoc test and presented as mean ± SEM. Scale bar = 20 μm. (n= 18 neurons/group). **P* < 0.05, ***P* < 0.01, and ****P* < 0.001.


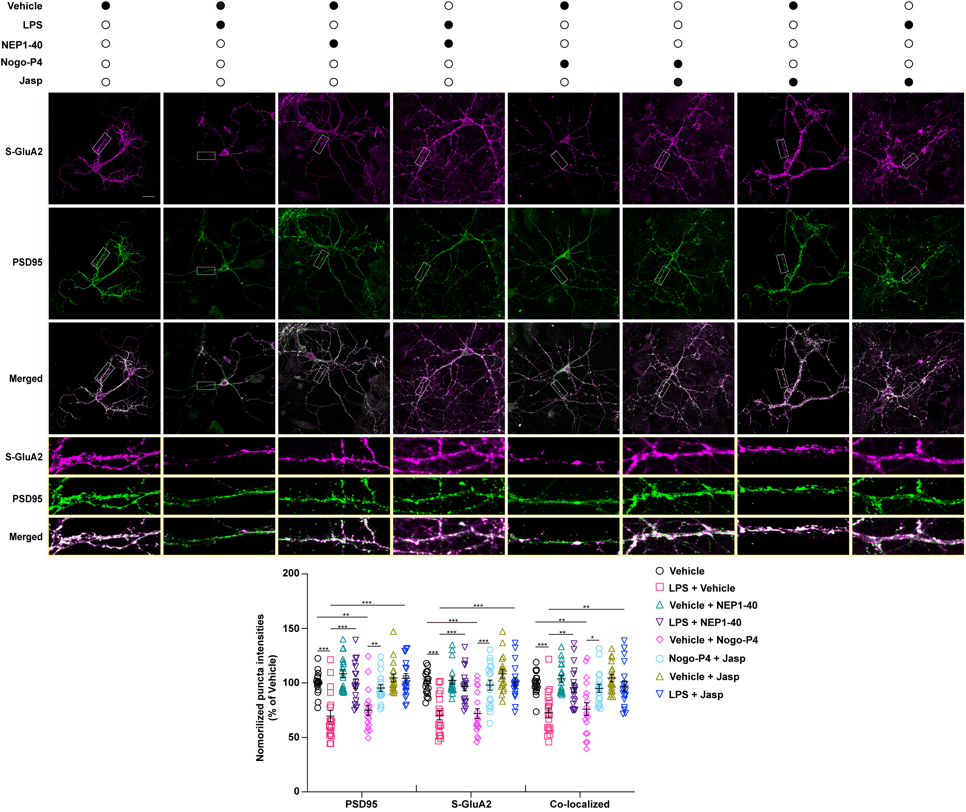


**Supplement 5. Expressions of PSD95 and surface GluA2 influenced by LPS, Nogo-P4, NEP1-40, and Jasplakinolide intervention on BV2-hippocampal neuronal co-cultures**

All data were analyzed using one-way ANOVA followed by Tukey’s post hoc test and presented as mean ± SEM (n= 18 neurons/group). Scale bar = 20 μm. **P* < 0.05, ***P* < 0.01 and ****P* < 0.001.


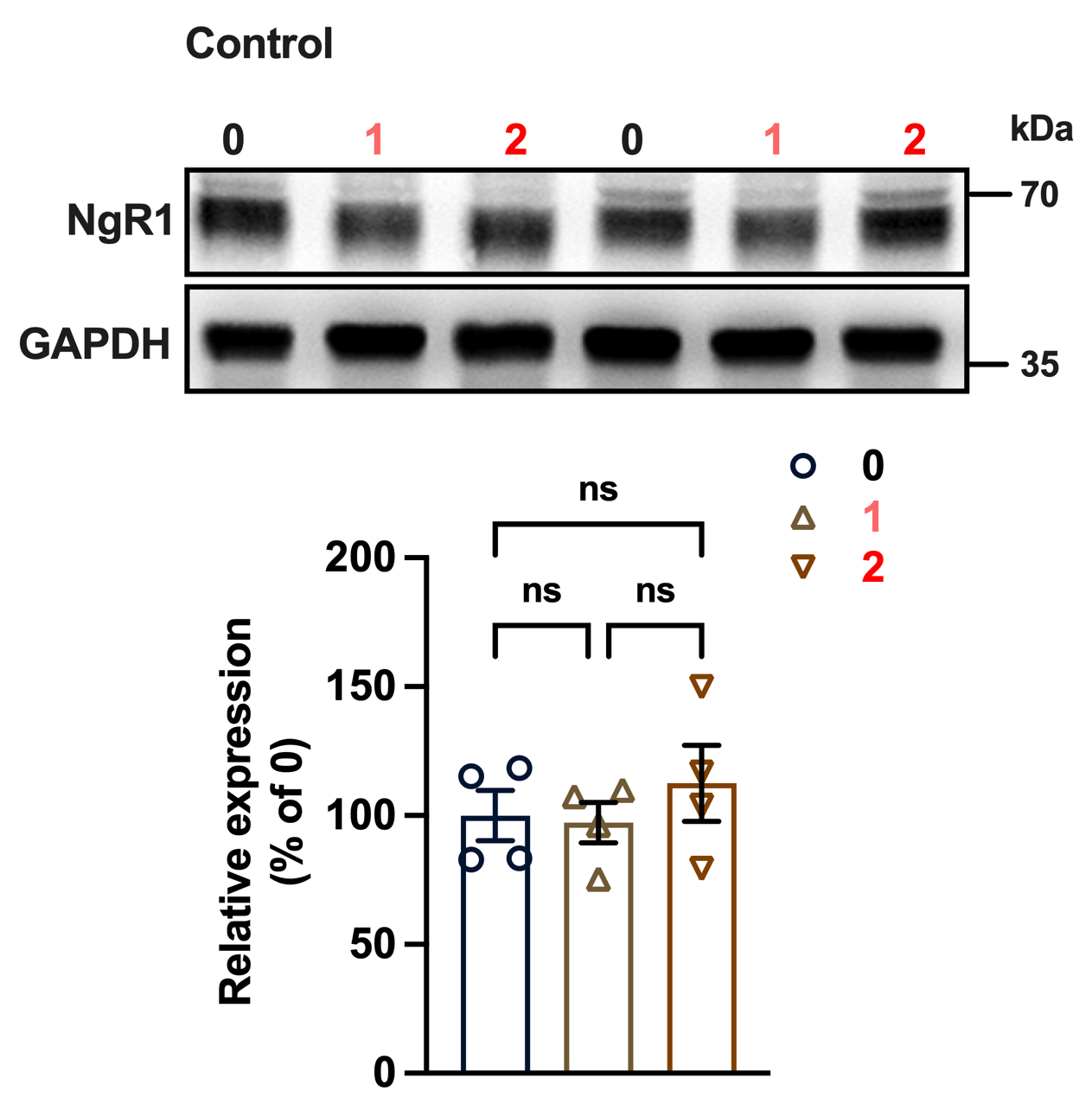


**Supplement 6. Expression of hippocampal NgR1 in the control group without any surgery, one-time, and two-time surgical procedures for osmotic minipump and optical fiber implantation before the anaesthesia and laparotomy surgery in aged mice**

All data were analyzed using one-way ANOVA followed by Bonferroni’s post hoc test and presented as mean ± SEM (n= 4/group). The group names of 0, 1, and 2 are represented as the control group without any surgery, one-time, and two-time surgery, respectively.
