# Supplementary figures and images for: Hippocampal Nogo66‐NgR1 signaling activation restricts postsynaptic assembly in aged mice with postoperative neurocognitive disorders
Source: Aging Cell. 2024 Oct 16;24(1):e14366. doi: 10.1111/acel.14366 (PMC11709113; doi:10.1111/acel.14366)

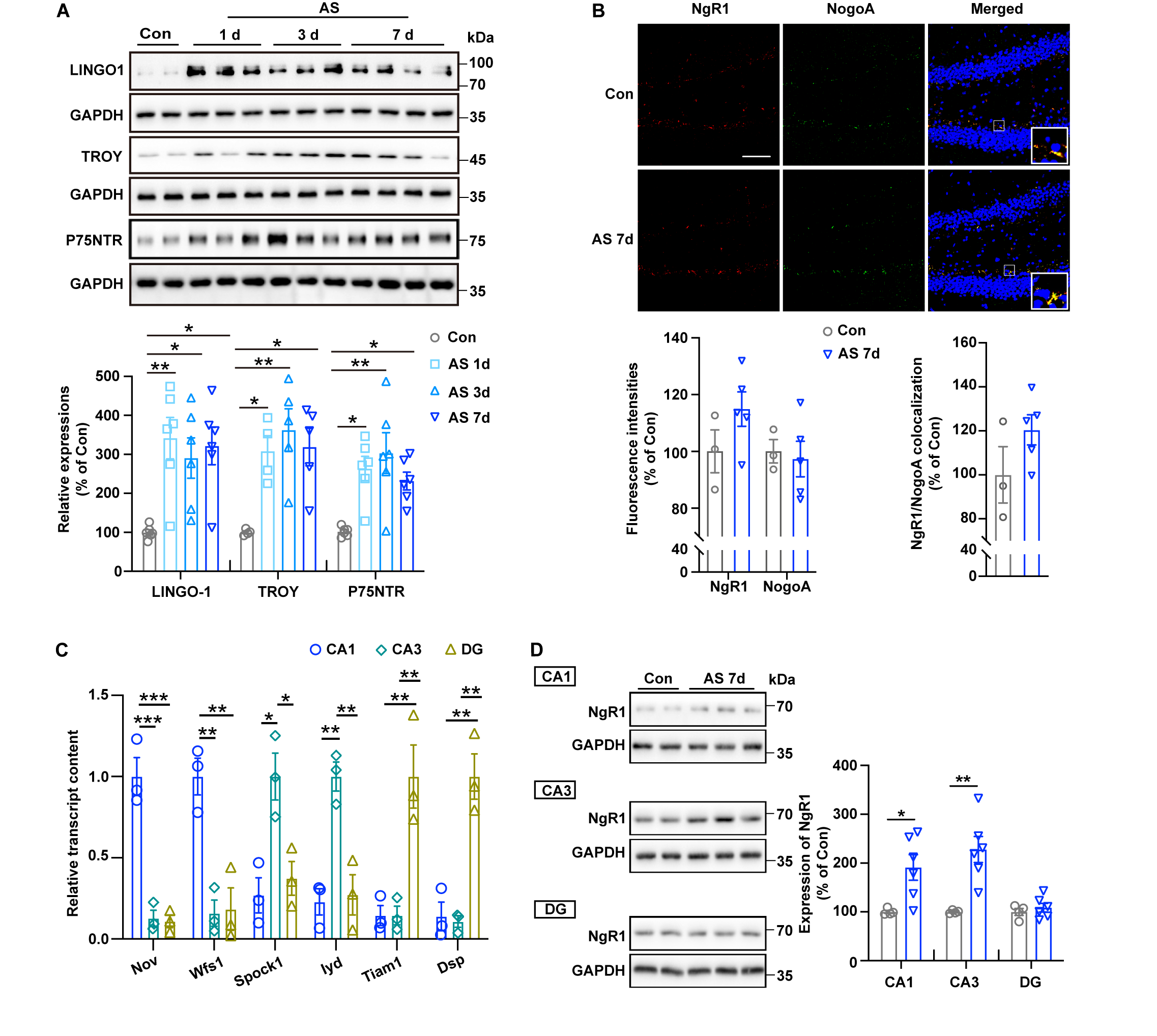

Supplement: Supplementary file 1 — Appendix S1. [file ACEL-24-e14366-s001.zip › Supplement 1.tif]

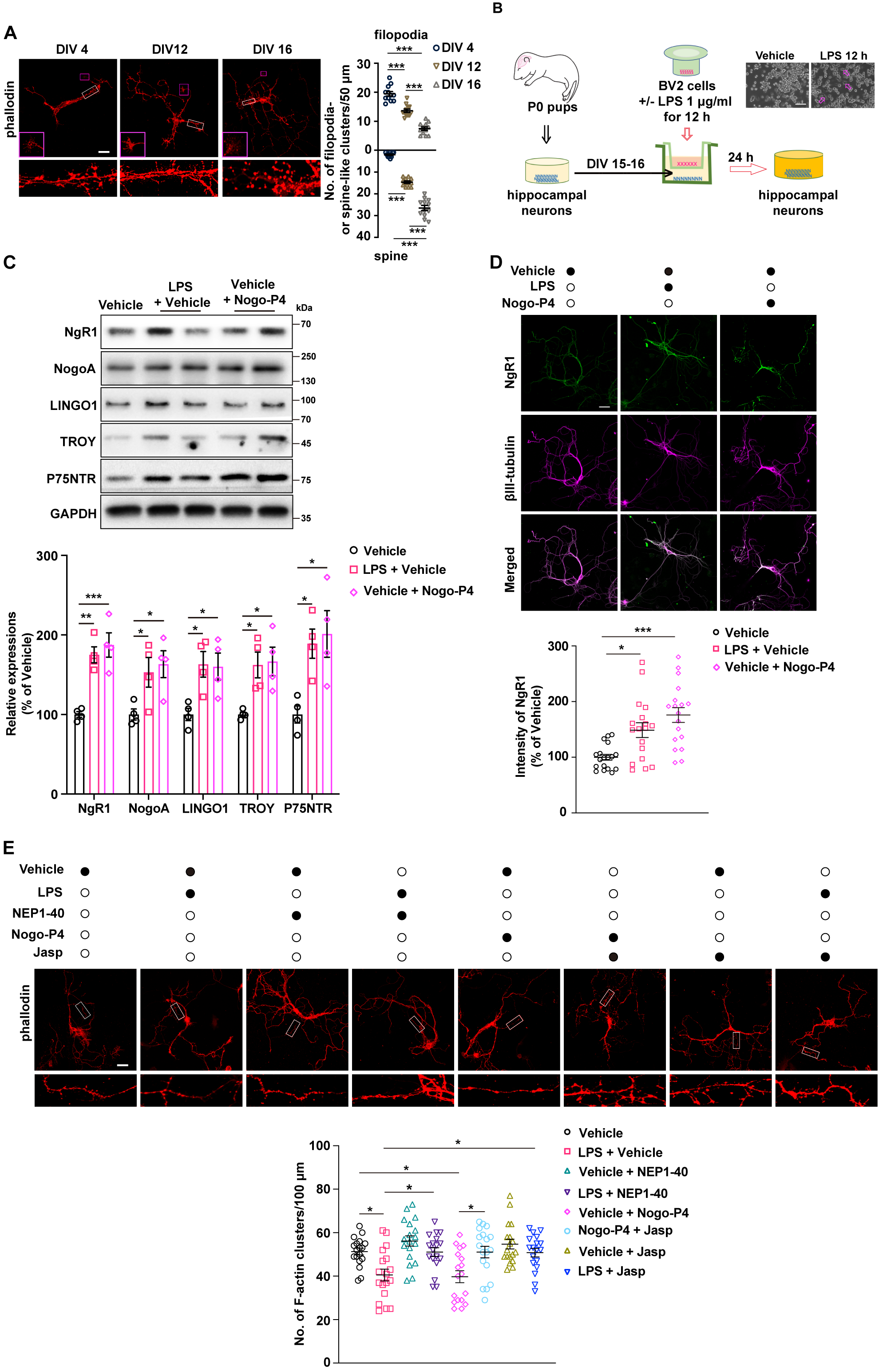

Supplement: Supplementary file 1 — Appendix S1. [file ACEL-24-e14366-s001.zip › Supplement 2.tif]

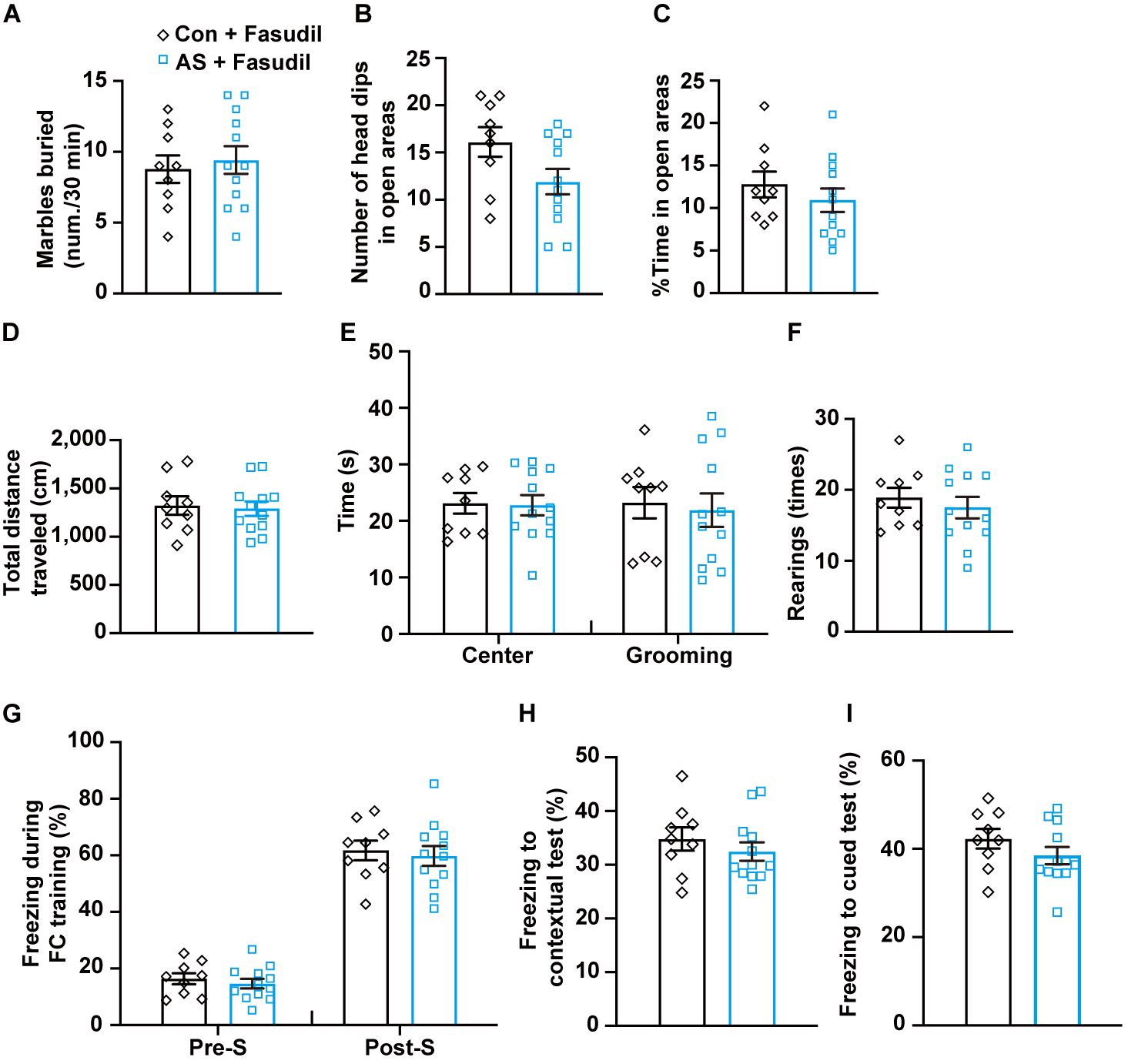

Supplement: Supplementary file 1 — Appendix S1. [file ACEL-24-e14366-s001.zip › Supplement 3.tif]

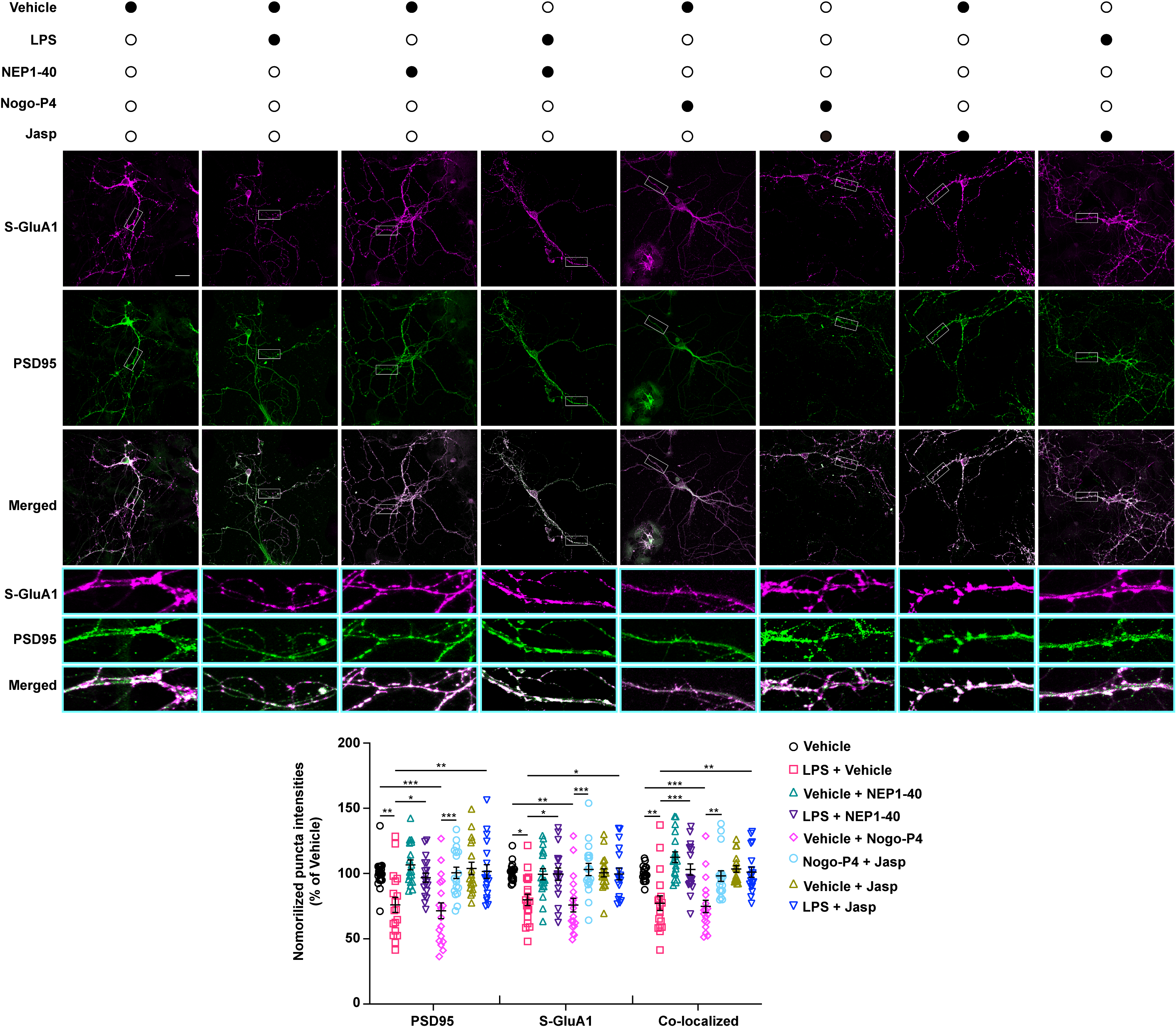

Supplement: Supplementary file 1 — Appendix S1. [file ACEL-24-e14366-s001.zip › Supplement 4.tif]

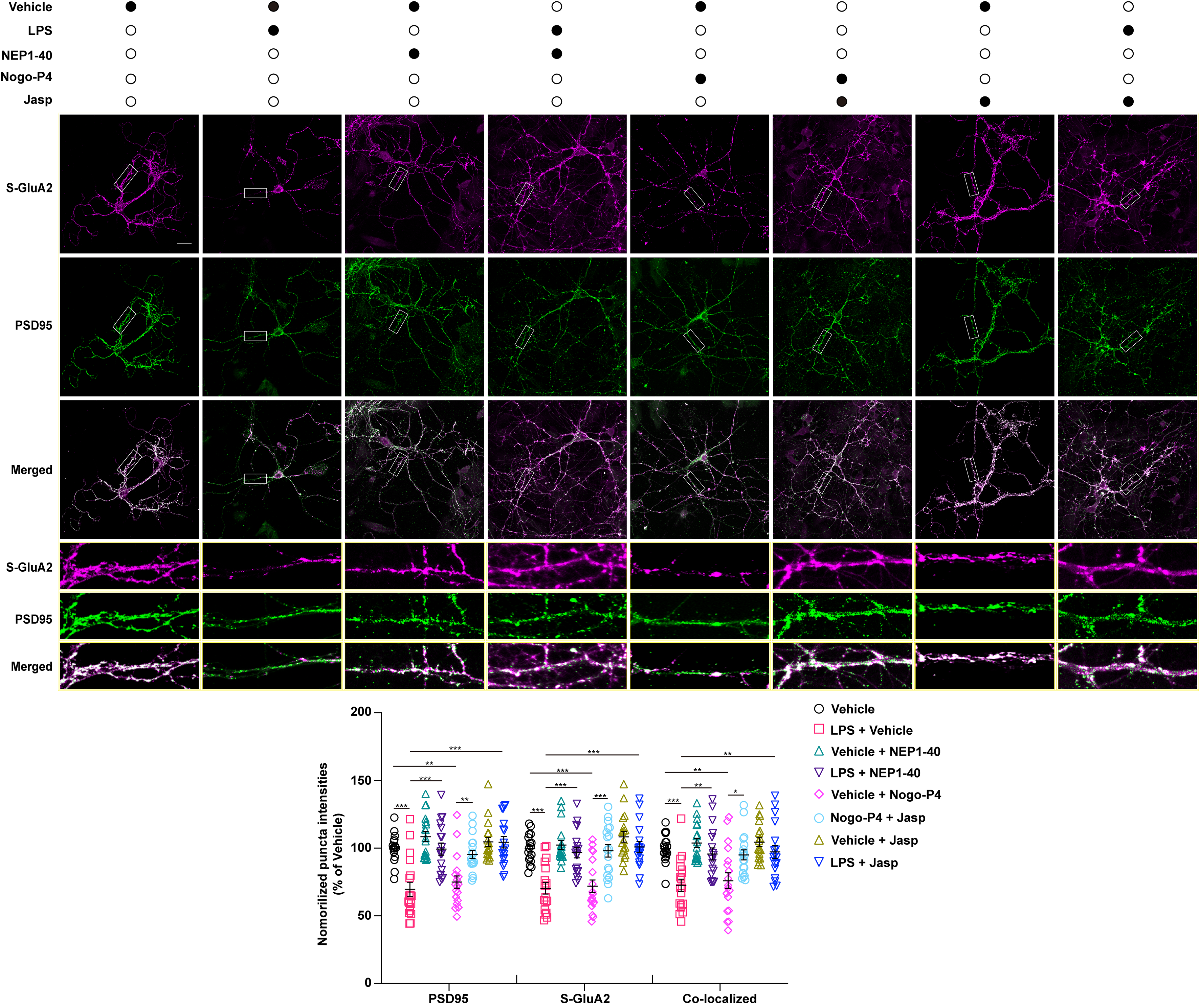

Supplement: Supplementary file 1 — Appendix S1. [file ACEL-24-e14366-s001.zip › Supplement 5.tif]

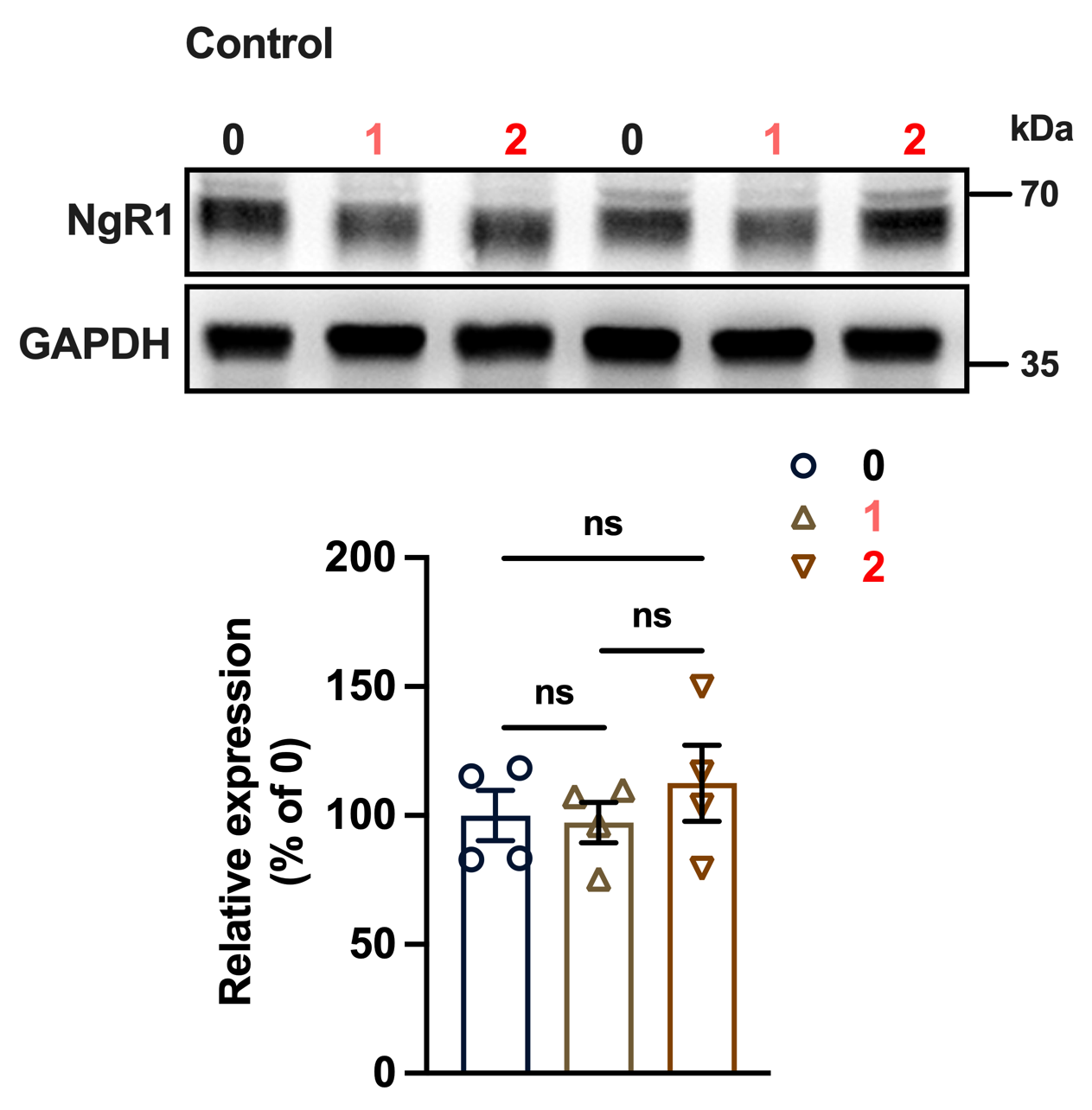

Supplement: Supplementary file 1 — Appendix S1. [file ACEL-24-e14366-s001.zip › Supplement 6.tiff]
